# Hyperspectral dual-comb compressive ghost imaging with deep learning reconstruction

**DOI:** 10.1038/s41377-026-02417-z

**Published:** 2026-09-16

**Authors:** Myoung-Gyun Suh, David Dang, Maodong Gao, Yucheng Jin, Dong-Chel Shin, Aggraj Gupta, Byoung Jun Park, Can Uzundal, Beyonce Hu, Wilton J. M. Kort-Kamp, Ho Wai Howard Lee

**Affiliations:** 1 Physics & Informatics Laboratories, NTT Research, Inc., Sunnyvale, CA USA; 2 Department of Physics & Astronomy, University of California Irvine, Irvine, CA USA; 3 Beckman Laser Institute and Medical Clinic, University of California Irvine, Irvine, CA USA; 4 Eddleman Quantum Institute, University of California Irvine, Irvine, CA USA; 5 Center for Integrated Nanotechnologies, Los Alamos National Laboratory, Los Alamos, NM USA; 6 Theoretical Division, Los Alamos National Laboratory, Los Alamos, NM USA

**Keywords:** Optical sensors, Imaging and sensing, Microscopy, Frequency combs

## Abstract

Ghost imaging reframes image formation by replacing pixelated sensors with correlation measurements between structured illumination and a single-pixel detector, enabling capabilities that are difficult or impossible for conventional cameras. This powerful technique is inherently suited to imaging through scattering media, photon-starved scenes, and size-, access-, or cost-constrained platforms. Yet its broader adoption has been limited by a fundamental speed bottleneck: slow, sequential pattern projection via spatial light modulators or wavelength sweeps, which precludes real-time capture of dynamic scenes. Here, we overcome this fundamental bottleneck by combining dual-comb interferometry with compressive ghost imaging and deep learning reconstruction. Our method, hyperspectral dual-comb compressive ghost imaging, utilizes optical frequency combs to create wavelength-multiplexed speckle patterns that are delivered through a single-core fiber and detected by a single-pixel detector. This approach enables the simultaneous illumination and detection of hyperspectral speckle patterns without slow spatial light modulation or wavelength scanning, thereby allowing snapshot compression and the acquisition of image information using spatially non-resolved hardware. To decode the compressed signals, we develop a transformer-based deep-learning model capable of rapid, high-fidelity image reconstruction at sampling ratios below 1%. Our hardware–software co-design approach achieves high-fidelity dynamic ghost imaging at video rates and beyond while dramatically simplifying the optical front end, significantly advancing ghost imaging toward practical deployment in size- and access-constrained scenarios—from minimally invasive endomicroscopy to compact portable sensors.

## Introduction

Ghost imaging has emerged as a powerful alternative to conventional imaging by reconstructing spatial information from correlations between known illumination patterns and single-pixel intensity measurements, rather than direct spatial sampling with detector arrays^1–9^. Beyond this conceptual novelty, the single-pixel detection approach offers distinct advantages in situations where multi-pixel sensors are difficult or impossible to use—for example, in low-light settings, through scattering media, or on platforms where hardware size is limited^10,11^. These benefits have motivated applications across diverse fields. For instance, in remote sensing, ghost imaging has been applied to imaging through turbulence and harsh atmospheric conditions where conventional cameras fail^3,12^. In security and defense, it enables imaging with lower optical visibility or even when there is no direct line of sight^13^. In biomedicine, ghost imaging has supported fluorescence and multiphoton microscopy in cases where detector arrays are too costly or not practical^14,15^. Quantum ghost imaging has even shown how entanglement can be used for secure imaging and information transfer, suggesting applications in future quantum communication networks^1,16^. Taken together, these wide-ranging demonstrations show that ghost imaging is not only an important new way of thinking about imaging, but also a flexible tool for real-world uses where robustness, compact design, or unconventional setups are needed (see Supplementary Section I for more background on ghost imaging).

Despite these advantages, traditional ghost imaging is fundamentally slow because it typically relies on spatial light modulators or swept-frequency lasers to generate uncorrelated 2D illumination patterns and project them onto the target sequentially. Due to the liquid-crystal or microelectromechanical operating principles of spatial light modulators, the pattern generation frame rate usually ranges from about 100 Hz to tens of kHz. The long measurement time required to project all illumination patterns therefore limits both speed and image quality, making it difficult to capture real-time images of dynamic events or moving objects^4,5,17,18^. Compressive sensing algorithms^19–22^ have helped reduce the number of patterns needed, but acquisition speed is still a major obstacle. Overcoming this challenge is essential to move ghost imaging beyond proof-of-concept experiments and toward practical applications requiring video-rate imaging.

Recent advances in optical frequency combs (OFCs)^23–25^ offer a pathway to parallelizing ghost imaging. OFCs are laser sources that emit a broad spectrum of mutually coherent, evenly spaced frequency lines, enabling wavelength-division multiplexed encoding and ultrafast acquisition across many channels simultaneously^26–32^. In particular, dual-comb interferometry provides a powerful method for parallel photodetection of all comb lines with a single detector, converting spectral modes into a temporal interferogram through multi-heterodyne detection^33–36^. While OFCs in conventional camera–based imaging are typically used to access additional imaging dimensions, such as hyperspectral imaging or depth-resolved 3D imaging^37,38^, recent demonstrations of dual-comb imaging or microscopy have achieved high frame rates and snapshot capabilities for 2D imaging^27,28,39,40^. However, these approaches often rely on bulky dispersive optics and direct pixel-by-pixel mappings, limiting both spatial resolution and system compactness.

**Fig. 1:**
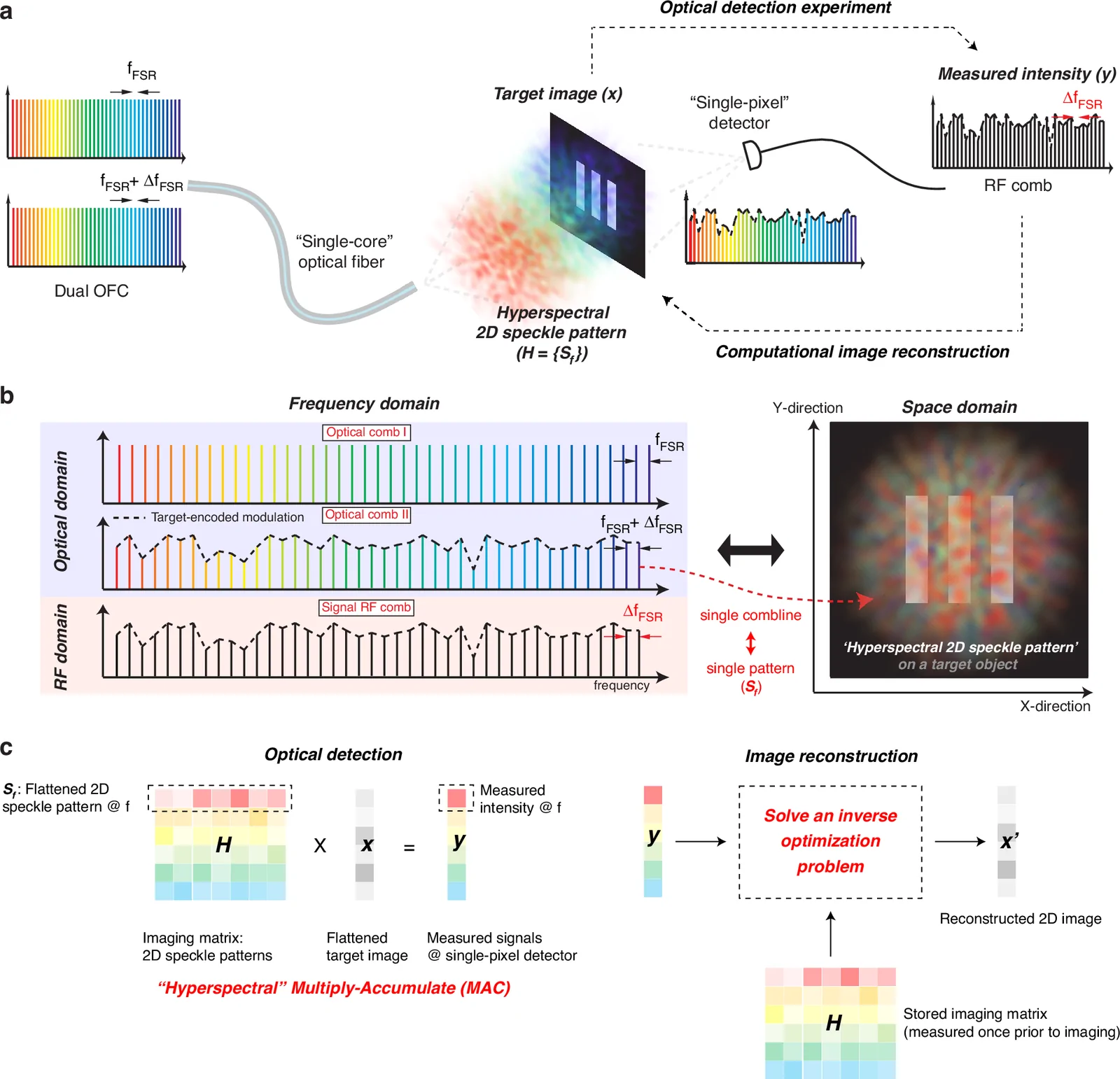
Hyperspectral dual-comb compressive ghost imaging. **a** Conceptual illustration of compressive ghost imaging with dual optical-frequency combs (OFCs). In the optical detection experiment, a hyperspectral speckle set *H* = {*S*_*f*_}—uncorrelated 2D speckle patterns *S*_*f*_ at each comb-line frequency—is generated at the fiber tip and mapped onto the target *x*. The encoded speckle pattern (*H* × *x*) is collected by a single-pixel detector, and the resulting dual-comb interference signal (“interferogram”) is recorded using an oscilloscope. A fast Fourier transform (FFT) converts this time-domain interferogram into a radio-frequency (RF) comb, yielding the bucket-sum signal (*y* = *H* × *x*) used for computational image reconstruction. With comb-based wavelength-division multiplexing (WDM) and dual-comb readout, the optical front end reduces to a single-core fiber and a single-pixel detector, enabling compact applications such as endomicroscopy. The experiment is performed in transmission, but a reflection configuration is also possible (see Supplementary Section II). **b** In our approach, each optical comb line generates an uncorrelated speckle pattern, enabling parallel optical encoding of target object information. The encoded optical modulation is then downconverted into the RF domain via dual-comb interferometry using two combs with slightly different line spacings, *f*_FSR_ and *f*_FSR_ + ∆*f*_FSR_, allowing parallel, high-SNR bucket-sum measurements for fast, high-fidelity imaging. In our experiment, Comb I and Comb II are combined before illuminating the target. Alternatively, one comb can probe the target and be recombined with the other afterward; the latter configuration is shown for clarity. **c** Simplified mathematical view of optical detection and computational image reconstruction processes. During optical detection, the 3D hyperspectral hypercube *H* (spanning frequency and 2D space) is multiplied by the 2D target *x*, producing 1D bucket-sum data *y* in the frequency domain. This operation is essentially a “hyperspectral” multiply-accumulate (MAC) process^41^. The pre-recorded speckle patterns *H* and measured *y* are then used to reconstruct *x* by solving an inverse optimization problem

In this work, we overcome these challenges by introducing hyperspectral dual-comb compressive ghost imaging, which merges dual-comb interferometry with ghost imaging principles and deep-learning–based reconstruction. By mapping frequency-comb lines to mutually uncorrelated two-dimensional speckle patterns and applying a dual-comb “hyperspectral” multiply–accumulate (MAC) reduction^41^, as illustrated in Fig. 1, we compress three-dimensional optical information (2D spatial × 1D spectral hypercube) into snapshot parallel intensity measurements acquired with a spatially non-resolved optical front end—a single-core illumination fiber and a single-pixel detector—thereby eliminating slow, sequential spatial pattern projection. To invert the compressed measurements, we develop Ghost-GPT, an application-specific deep-learning transformer model^42^ that achieves high-fidelity reconstructions and enables video-rate imaging of dynamic targets. This hardware–software co-design not only removes the speed bottleneck of conventional ghost imaging but also enables a compact, low-cost front end, making it well suited to constrained settings such as minimally invasive endomicroscopy^43–52^ and bringing ghost imaging closer to practical deployment.

Notably, unlike other spectral correlation–based ghost imaging using swept-frequency lasers^53,54^, our dual-comb approach allows fundamentally parallel pattern generation, which is crucial for imaging moving targets, as sequential pattern projection can introduce motion artifacts. Moreover, the inherent accuracy and stability of optical frequency combs ensure repeatable speckle patterns at each comb line. We believe that this combination of parallel and repeatable pattern generation is essential for practical video-rate ghost imaging.

## Results

Figure 1a illustrates the concept of our hyperspectral dual-comb compressive ghost imaging method, which utilizes combline-to-speckle pattern mapping (Fig. 1b). The method consists of two main steps: the optical detection experiment and computational image reconstruction (Fig. 1c).

In the optical detection experiment, a set of speckle patterns *H* = {*S*_*f*_}, consisting of uncorrelated 2D speckle patterns *S*_*f*_ at different comb line frequencies *f*, is generated at the fiber tip using a dual-OFC source and projected onto the 2D target image *x*. The encoded intensity distribution (*H* × *x*) is detected by a single-pixel photodetector. Because each comb source has a slightly different free spectral range (FSR), *f*_FSR_ and *f*_FSR_ + ∆*f*_FSR_, the resulting time-varying voltage signal, containing the dual-comb interference (“interferogram”), is recorded with an oscilloscope. A fast Fourier transform (FFT) converts this time-domain signal into radiofrequency-domain bucket-sum data, enabling parallel and precise electronic detection of *y* = *H* × *x*. At a high level, this information processing approach achieves data dimensionality reduction through a hyperspectral MAC operation^41^, as well as hardware dimensionality reduction through wavelength-division multiplexing (WDM) and dual-comb readout—enabling minimal front-end imaging hardware and significantly increasing imaging speed. The mutual coherence of the dual combs allows coherent averaging to improve the signal-to-noise ratio (SNR)^35^, which is another key advantage of the dual-comb method.

**Fig. 2:**
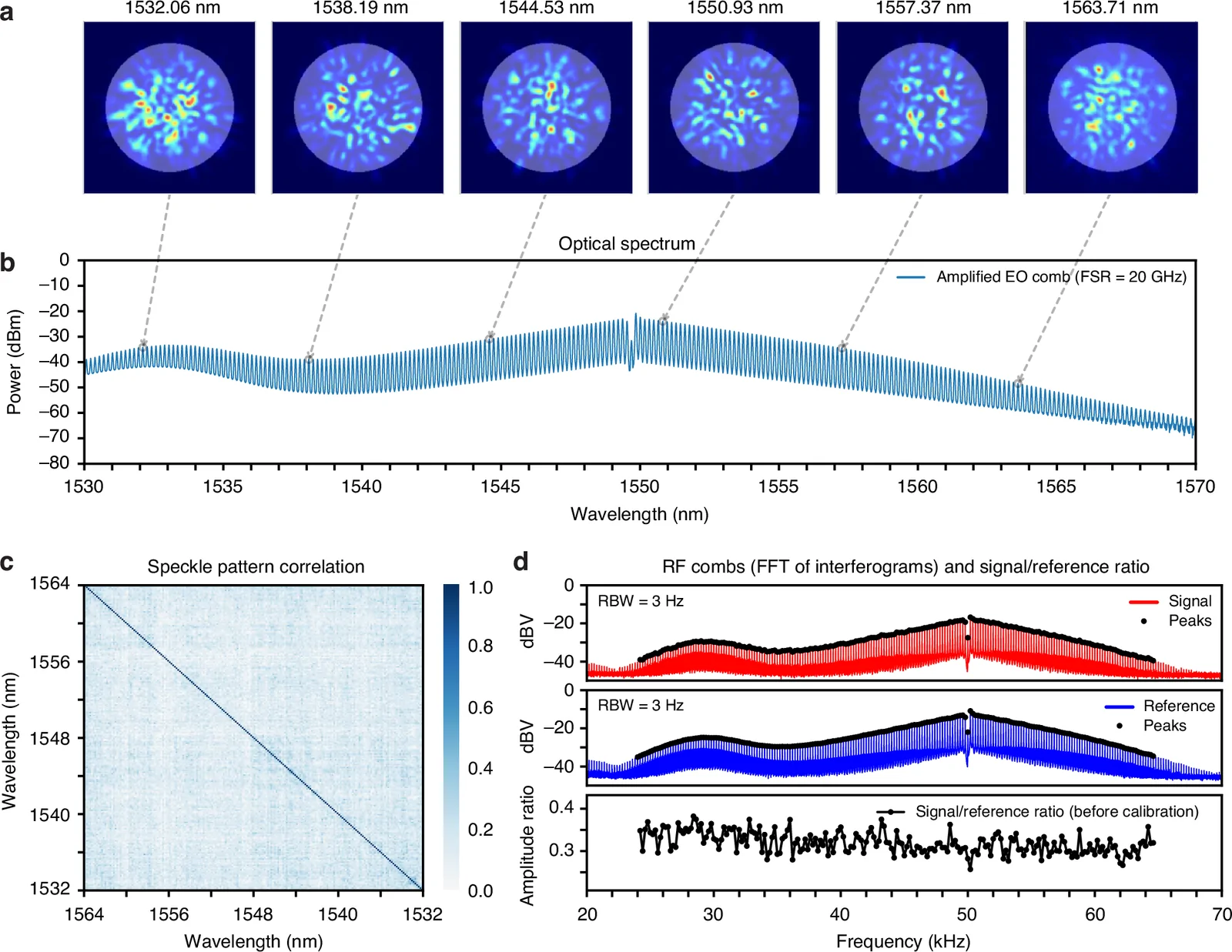
Experimental details. **a** Speckle patterns measured at six different comb line frequencies. Each image contains 256 × 256 pixels and exhibits a circular speckle pattern centered within the frame. **b** Typical optical spectrum of the electro-optically (EO) modulated comb with a 20 GHz free spectral range (FSR). The EO comb is generated from a 1550 nm continuous-wave (CW) fiber laser via resonant EO modulation and subsequently amplified by an erbium-doped fiber amplifier (EDFA) before being coupled into the experimental setup. **c** Pearson correlation coefficients calculated between speckle patterns across the wavelength range from 1532 nm to 1564 nm. The correlation is computed within the bright circular region of each speckle pattern. The low off-diagonal values indicate that the speckle patterns are largely uncorrelated. Residual correlations are attributed primarily to the common background near the edges of the speckle patterns. **d** Typical radio-frequency (RF) comb spectra generated from dual-comb interference signals, as measured by the signal and reference single-pixel detectors (upper and middle panels). The ratio of the comb peak amplitudes between the signal and reference RF combs is shown in the bottom panel. Differences between the signal and reference beam paths are calibrated using measurements performed with and without the target object. RBW resolution bandwidth

As shown in Fig. 2, we first imaged static USAF 1951 target patterns using approximately 200 comb lines from each of the 20 GHz electro-optic (EO) combs spanning 1532–1564 nm (Fig. 2b), with Δ*f*_FSR_ = 200 Hz. The speckle patterns (*H*) without the target were recorded using a 2D InGaAs camera by selecting individual comb lines via the Waveshaper, either before or after imaging the target. The speckle patterns recorded at different comb-line frequencies exhibit distinct spatial distributions (Fig. 2a), and Pearson correlation analysis confirms that the speckle patterns are largely uncorrelated with 20 GHz spacing (Fig. 2c). Residual correlations are mainly attributed to common dark background components, especially near the pattern edges. Each comb line has a different power level, so the speckle patterns are normalized accordingly. To extract the target image information and suppress temporal noise, simultaneous dual-comb measurements are performed in both the signal path containing the target object and the reference path without the target. Figure 2d shows the bucket-sum measurements obtained from the signal and reference RF combs (via FFT of the interferograms), along with their ratio, which contains the encoded image information and is used for image reconstruction. The same measurement is also performed without the target object and is used to calibrate optical path imbalances between the signal and reference arms (see Supplementary Section II for more experimental details).

**Fig. 3:**
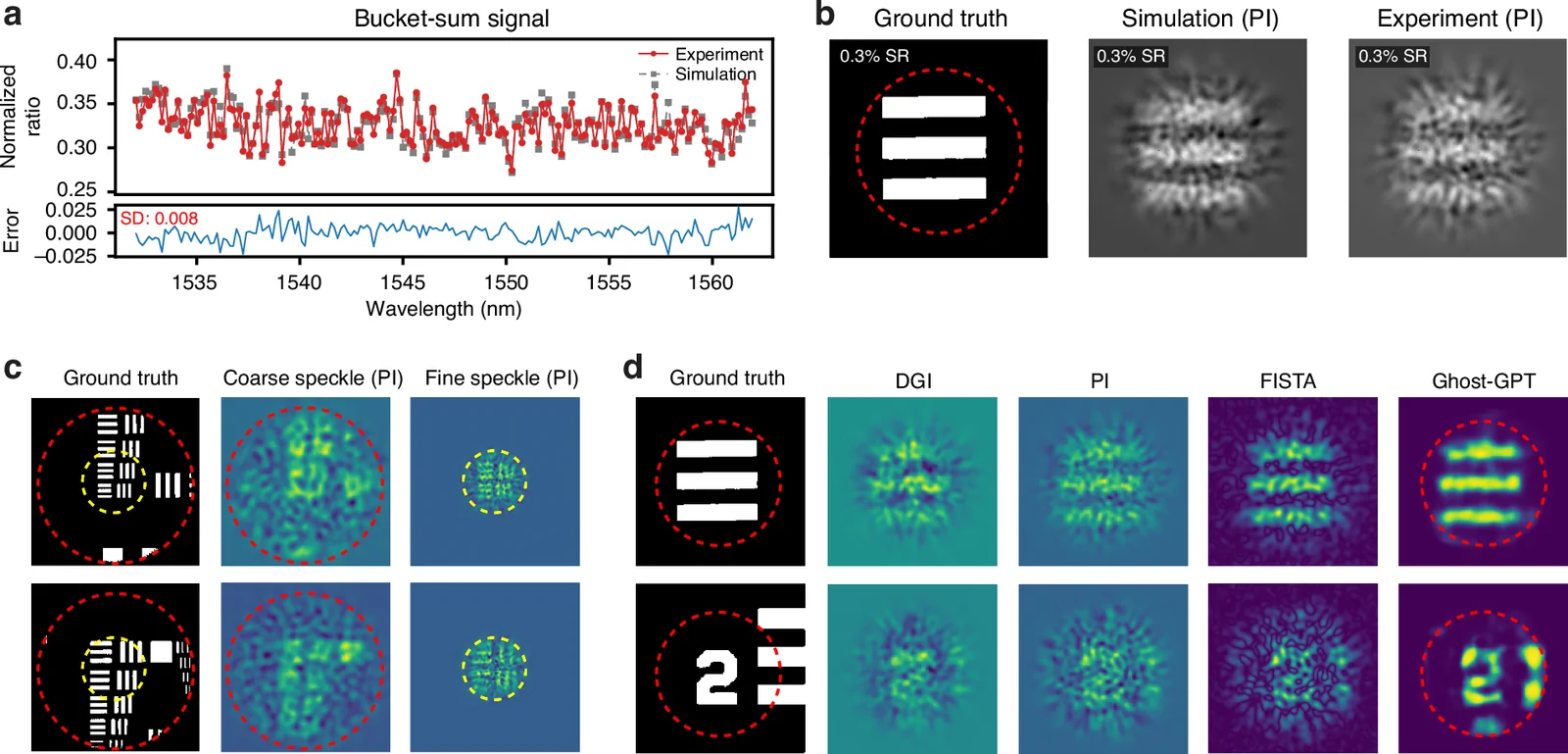
Image reconstruction of static targets. **a** The calibrated bucket-sum signal measured in the dual-comb experiment shows excellent agreement with the simulated bucket-sum signal, obtained by applying the ground-truth mask (USAF 1951 resolution target) to the stored speckle-pattern images; the standard deviation between the two signals is 0.008. **b** Image reconstruction is performed using the Moore–Penrose pseudoinverse (PI) algorithm at a sampling ratio (SR) of 0.3%. Both the simulation (left) and the experiment (right) successfully recover the ground-truth patterns, with residual background noise arising from the illumination speckle. **c** Image reconstruction of smaller patterns in group 2 (4.00 - 7.14 lp/mm). Fine details cannot be resolved when using a large illumination area with coarse speckle grains, but become resolvable with fine speckle grains when the beam is focused on a smaller area. Red and yellow dotted circles indicate the illuminated areas in each case. **d** To improve reconstructed image quality, we developed a transformer-based deep learning model, Ghost-GPT. Reconstructions from experimental bucket-sum measurements using Ghost-GPT are compared with classical algorithms: Differential Ghost Imaging (DGI), Moore–Penrose Pseudoinverse (PI), and the Fast Iterative Shrinkage-Thresholding Algorithm (FISTA). (Top row): Striped lines correspond to group 0, element 4 (1.41 line pairs per millimeter). (Bottom row): The number 2 corresponds to group 0, element 2 (1.12 lp/mm)

For computational image reconstruction, we used the calibrated bucket-sum signals together with prerecorded speckle patterns. Figure 3a shows the calibrated bucket-sum signals, in which the dual-comb measurements agree well with the simulated prediction. The reconstructed image using the Moore–Penrose pseudoinverse (PI) algorithm clearly reveals the object (Fig. 3b). However, the reconstruction exhibits significant speckle-induced background noise. As an aside, speckle patterns generated by different media (e.g., multimode fibers with various core sizes) display different speckle grain sizes, corresponding to distinct narrowband spatial frequencies. To improve image resolution and fidelity, several approaches can be considered to generate speckle patterns with broader spatial frequency content. For example, the speckle pattern generator could be designed such that speckle patterns from different comb lines have different grain sizes, and thus different spatial frequencies. Additionally, as shown in Fig. 3c, adjusting the beam spot size on the object can effectively modify the spatial frequency of the speckle patterns and enhance image resolution.

To further enhance image quality, we developed a transformer-based image reconstruction algorithm^55,56^ that significantly reduces background noise. The model, which we call Ghost-GPT, is tailored for ghost imaging applications. While Generative Pretrained Transformers (GPTs) are commonly associated with natural language processing (e.g., ChatGPT) and human text, they are general-purpose architectures that can also process images, time-series information, or other structured data types. Hence, we adopt the name of “Ghost-GPT” to reflect our use of a GPT-style architecture for processing the speckle patterns and bucket measurements of ghost imaging. The model compresses the speckle patterns into a latent space and concatenates them with the corresponding bucket measurements to form the input token sequence. This sequence is then processed through a stack of 12 transformer blocks, each comprising a multihead self-attention (MHSA) mechanism and a two-layer feedforward network. The model outputs a reconstructed image with a resolution of 256 × 256 pixels.

For training, we generate a large dataset of simulated bucket measurements by convolving our experimentally measured wavelength-dependent speckle patterns with known image datasets (e.g., MNIST and Omniglot). The model learns the mapping between the speckle-bucket measurement pair and the corresponding ground-truth image by backpropagating the error between the predicted image and the true image from the dataset. The trained model is then directly applied to experimentally acquired bucket signals with our optical setup for image reconstruction. A detailed description of the model architecture and performance analysis is provided in the Supplementary Section III.

Using the Ghost-GPT model with our optics setup, we were able to reconstruct high-fidelity images, achieving a Structural Similarity Index Measure (SSIM) greater than 0.6 and a Mean Squared Error (MSE) less than 0.05, despite a sampling ratio (number of illumination patterns divided by the number of pixels in the reconstructed image) of only 0.3% (see Table 1 and Fig. 3d). Here, SSIM quantifies the perceived quality of the reconstructed image relative to the ground truth image by comparing their luminance, contrast, and structure (higher SSIM is better)^57^. We also include MSE as another complementary metric, as it measures the raw pixel-wise error between the images (lower MSE is better). The model effectively preserves fine structural details and clear object boundaries while suppressing background noise, highlighting its ability to recover meaningful image content from sparse measurements. We also compared our model with classical and other deep-learning reconstruction algorithms and found that Ghost-GPT generally outperforms them in both speed and reconstructed image fidelity (see Table 1 and Supplementary Section III). In particular, Ghost-GPT generally outperforms classical algorithms by roughly an order of magnitude difference in SSIM. The model’s rapid reconstruction speed of 14 ms enables real-time video-rate ghost imaging in optical fibers. Importantly, while the computational reconstruction speed can be further improved with more powerful computing hardware, the fundamental frame-rate limit is set by ∆*f*_*FSR*_ of the dual comb, which in our experiments ranges from a few hundred Hz to several kHz.

**Table 1:** MSE/SSIM for experimental targets with classical algorithms and Ghost-GPT

Target	DGI	PI	FISTA	Ghost-GPT (Ours)
Stripes	0.231/0.030	0.140/0.028	0.057/0.097	**0.045/0.560**
Number 2	0.204/0.036	0.138/0.028	0.070/0.113	**0.053/0.634**

Bold numbers highlight the lowest MSE and the highest SSIM (i.e. the best resulting metrics from all of the reconstruction techniques)

**Fig. 4:**
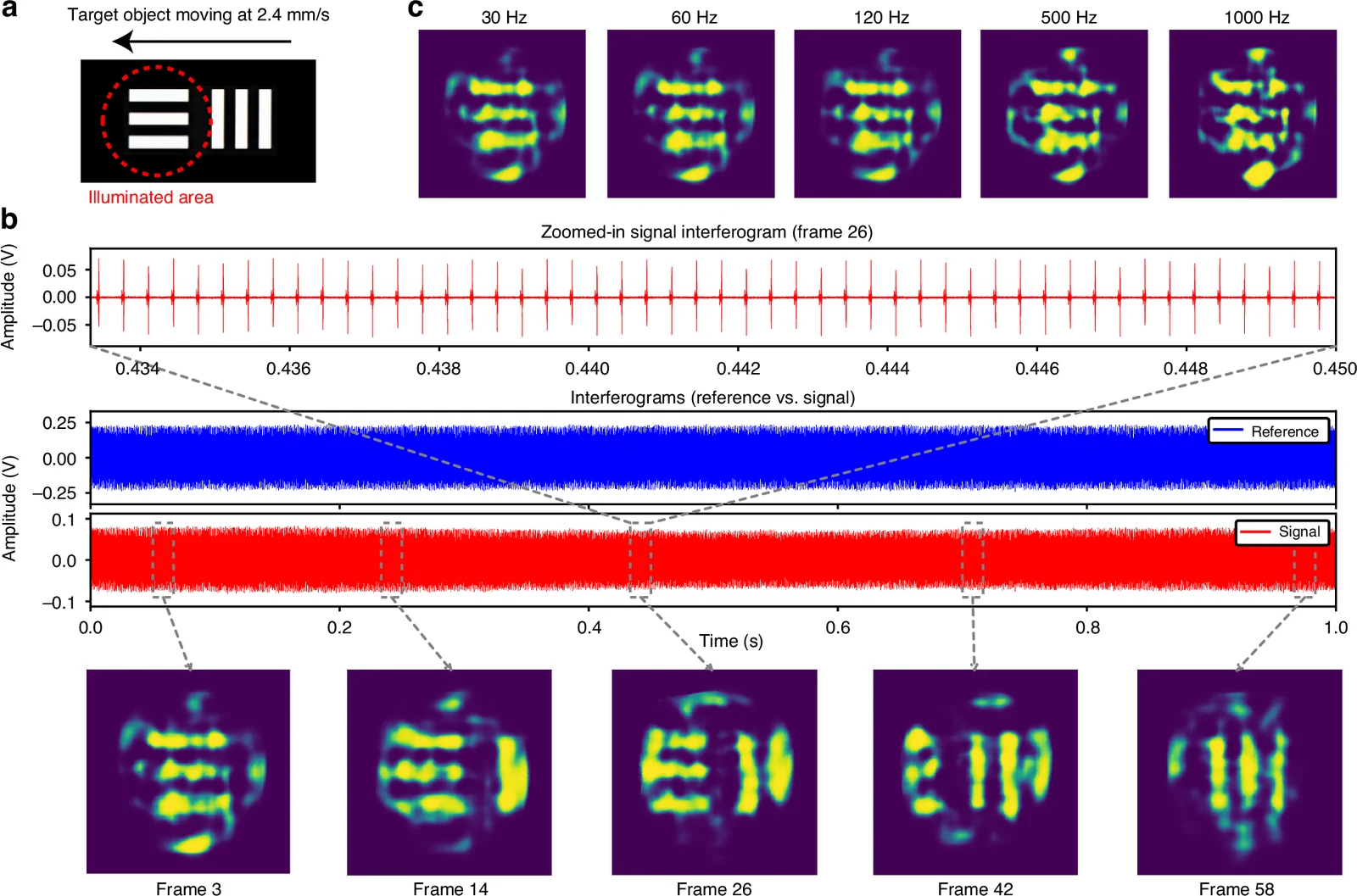
Video-rate image reconstruction of a moving target. **a** A hyperspectral speckle pattern is projected onto a USAF 1951 resolution target (Group 0, Element 4; 1.41 lp/mm), which is mounted on a motorized stage and translated horizontally at a speed of 2.4 mm/s (the maximum speed of the stage). **b** Reference and signal interferograms of the moving object acquired over 1 second (middle panel), along with a zoomed-in view of the signal interferogram (top panel) used to reconstruct frame 26. The bottom panel displays five reconstructed images at a 60 Hz frame rate, capturing the object’s motion. **c** Reconstructed images of the object at t = 50 ms (corresponding to the start time of frame 3), obtained from the interferograms in (**b**), at five different frame rates (f = 30, 60, 120, 500, and 1000 Hz). Given the dual-comb repetition rate difference of 3 kHz, each image is reconstructed from 3000/f electrical pulses. The results demonstrate that imaging a much faster moving target is feasible

To demonstrate the high-speed imaging capability of our approach, we set ∆*f*_*FSR*_ = 3 kHz and imaged a target pattern moving at 2.4 mm/s (see Fig. 4). For video-rate imaging at a 60 Hz frame rate, the 1-second-long signal and reference interferograms (Fig. 4b, middle panel) were divided into 60 time intervals. Each interval (Fig. 4b, top panel) was Fourier-transformed to extract the bucket-sum signal. The Ghost-GPT model was then used to reconstruct images for each interval (frames 0–59), with five representative frames shown in the bottom panel of Fig. 4b. At ∆*f*_*FSR*_ = 3 kHz, higher frame rates of up to 1 kHz were achievable, though with slight degradation in the SNR of the bucket-sum signals and in the quality of the reconstructed images (Fig. 4c).

## Discussion

The demonstrated image quality and frame rate can be further improved with future system developments. In our current experiments, we were limited to using approximately 200 speckle patterns with comb spacings of 20 GHz or 25 GHz, constrained by the full-width at half-maximum (FWHM) of speckle pattern correlation and the line-by-line spectral filtering resolution of the Waveshaper used to resolve individual speckle patterns. Although this small number of speckle patterns restricted Ghost-GPT to tests on simple binary datasets, it is not a fundamental limitation. In principle, sub-GHz FWHM speckle correlation is achievable^58,59^, and speckle pattern acquisition at such narrow comb spacings could be realized using a tunable CW laser calibrated against the comb^60^. Combined with spectral broadening of OFCs, this approach would enable the use of a substantially larger number of speckle patterns for image reconstruction, thereby allowing evaluation on more realistic grayscale datasets and improving both fidelity and resolution^56^ (see Supplementary Section III.J for a numerical simulation of ghost imaging with natural grayscale images).

For instance, with a 1 GHz comb spacing across a 100 nm spectral span near 1550 nm (yielding approximately 12,500 comb lines), a ∆*f*_*FSR*_ of 10 kHz, and a sampling ratio (SR) of 1%, high-definition (HD) resolution video-rate imaging could be achieved with an effective fill rate of 12 gigapixels per second (Fill rate = ∆*f*_*FSR*_*/M* × *N* × 1*/SR*). Here, the frame rate (∆*f*_*FSR*_*/M*) is determined by the free spectral range difference divided by the number of averaging periods *M* required to enhance SNR. Currently, our system’s frame rate is primarily limited by the photodetector bandwidth (*f*_*BW*_) and the number *N* of radiofrequency comb lines, governed by the condition *N*× ∆*f*_*FSR*_ < *f*_*BW*_. In principle, both the frame rate and the sampling ratio can be flexibly tuned depending on application requirements—balancing frame rate against image fidelity within the detector bandwidth limit.

The transformer-based deep-learning model used for image enhancement can be further optimized, but improvements in the raw data quality—such as using low-noise photodetectors or low-noise cameras for speckle pattern recording—would significantly enhance the quality of reconstructed images. System performance could also benefit from operating within wavelength ranges detectable by high-speed, high-sensitivity CMOS image sensors and photodetectors. Squeezed dual-comb sources offer an additional opportunity to improve performance by boosting the SNR and thereby reducing the required averaging time^61^. Although our current demonstration is limited to 2D spatial imaging, the same system is capable of operating in spectroscopic imaging or spectroscopy-only modes, depending on the application^38^. Furthermore, combining our spatial-domain compressive imaging with time-domain compressive sensing using time-programmable frequency combs^62,63^ would be an intriguing direction for future research, enabling simultaneous compression of the object’s four-dimensional hyperspectral spatio-temporal information across both spatial and temporal domains.

In conclusion, we experimentally demonstrate hyperspectral dual-comb compressive imaging that achieves high-speed ghost imaging with a minimal optical front end—a single-core fiber and a single-pixel photodetector. This simplicity is particularly important for hardware-constrained applications such as compact, cost-effective, single-use endomicroscopic probes. Furthermore, we show that image quality can be significantly enhanced by the transformer-based reconstruction model proposed in this study, effectively minimizing information loss during the compression process. While several technical improvements are still needed for practical applications— such as developing compact and reliable speckle-pattern (or structured-light) generators^53,64^, efficiently collecting light on the photodetector, and achieving grayscale imaging^56^—we present a key proof-of-concept demonstration showing that high-fidelity ghost imaging at or beyond video rate is possible even with minimally invasive, spatially non-resolved hardware configurations. This hardware–software co-design—comb-based parallel optical processing paired with deep-learning-enabled computational ghost imaging—holds strong promise for sensing and biomedical applications constrained by hardware size and complexity.

## Materials and methods

For the dual-comb source, we use two EO combs with slightly different free spectral ranges (FSRs). A continuous-wave (CW) fiber laser at 1550 nm is first amplified and split into two beams via a 50/50 fiber coupler. Each beam is frequency-shifted using an acousto-optic frequency shifter, introducing a center frequency offset ∆*f*_center_. These beams are then independently modulated by resonant EO modulators driven at *f*_FSR_ and *f*_FSR_ + ∆*f*_FSR_, respectively, where *f*_FSR_ = 20 GHz and ∆*f*_FSR_ = 200 Hz for the static object imaging experiment (or *f*_FSR_ = 25 GHz and ∆*f*_FSR_ = 3 kHz for moving targets). The resulting EO combs are recombined using another 50/50 fiber coupler and amplified to compensate for the insertion loss of the downstream system. Before entering the free-space imaging setup, a Waveshaper is placed in the system to optionally filter individual comb lines. For bucket-sum measurements, the full dual-comb spectrum is transmitted through the Waveshaper. Downstream, the 5-meter multimode fiber (MMF) used in the experiments has a core diameter of 200 microns and supports hundreds of spatial modes, resulting in distinct speckle patterns when light is outcoupled into the free-space setup. The generated speckle patterns are collimated and passed through the target object, a negative USAF 1951 resolution target mounted on a motorized translation stage. A 50/50 beam splitter divides the beam into signal and reference paths before the target, allowing simultaneous acquisition of signal and reference interferograms and thereby suppressing common-mode temporal noise. Both the reference and signal bucket-sums are acquired using free-space InGaAs photodetectors with a 510 kHz bandwidth. The reference path is in free space, although it could alternatively be implemented in fiber using a 50/50 fiber coupler, which may be more practical for real-world applications. Additionally, the imaging experiment can be conducted in reflective mode rather than the current transmissive configuration. The speckle pattern set (*H*) without the object is separately recorded using a 2D InGaAs camera by selecting individual comb lines with the Waveshaper, either before or after imaging. It is worth noting that the MMF was fixed to the optical table, and the resulting speckle patterns remained stable enough to support multiple imaging experiments over several hours. (Details of the experimental setup are provided in the Supplementary Information.)

## Supplementary information

Supplementary Information

## Data Availability

The data and code that support the plots within this paper and other findings of this study are available on our repository: [Repository Link].
