# Supplementary material for: Hyperspectral dual-comb compressive ghost imaging with deep learning reconstruction
Source: Light Sci Appl. 2026 Sep 16;15:380. doi: 10.1038/s41377-026-02417-z (PMC13578236; doi:10.1038/s41377-026-02417-z)
Supplement: Supplementary file 1 [file 41377_2026_2417_MOESM1_ESM.pdf]

Supplementary Information for "Hyperspectral dual-comb compressive ghost imaging with deep learning reconstruction"

Myoung-Gyun Suh[1*†], David Dang[2,3,4,5,6†], Maodong Gao[1†], Yucheng Jin[2,3,4], Dong-Chel Shin[1], Aggraj Gupta[1], Byoung Jun Park[1], Can Uzundal[1], Beyonce Hu[2,3,4], Wilton J.M. Kort-Kamp[6] and Ho Wai (Howard) Lee[2,3,4*]

[1] *Physics & Informatics Laboratories, NTT Research, Inc., Sunnyvale, CA 94085, USA.*
[2] *Department of Physics & Astronomy, University of California Irvine, Irvine, CA 92697, USA.*
[3] *Beckman Laser Institute and Medical Clinic, University of California Irvine, Irvine, CA 92617, USA.*
[4] *Eddleman Quantum Institute, University of California Irvine, Irvine, CA 92697, USA.*
[5] *Center for Integrated Nanotechnologies, Los Alamos National Laboratory, NM 87545, USA.*
[6] *Theoretical Division, Los Alamos National Laboratory, NM 87545, USA.*
[*] *Corresponding author. Email: myoung-gyun.suh@ntt-research.com, Howardhw.lee@uci.edu*
[†] *These authors contributed equally to this work.*

This PDF file includes:

FIG. S1 - S11
TABLES S1 - S3

CONTENTS

I. Background on Ghost Imaging 2

II. Experimental Details 3
- A. Dual Electro-Optic Comb Source 3
- B. Free-Space Imaging Setup 3
- C. Speckle Basis Acquisition 3
- D. Spectral Correlation Linewidth Characterization 3

III. Ghost-GPT: Transformer-based Image Reconstruction Model 5
- A. Model Architecture. 5
- B. Ghost-GPT Reconstruction Results 6
- C. Robustness of Ghost-GPT in Experimental Imaging 7
- D. Dataset Generation, Train and Test Split 7
- E. Hyperparameter Analysis 7
- F. Different Algorithms vs Signal-to-Noise Ratio 9
- G. Resolution Analysis 9
- H. Comparison with Other Deep Learning Models 9
- I. Experimental Hyperparameter Tuning 11
- J. Ghost Imaging of Natural Images 12

References 13

I. BACKGROUND ON GHOST IMAGING

The most straightforward form of single-pixel imaging involves raster scanning, where a single-pixel detector sequentially measures light intensity at each pixel location, requiring N^2 measurements for an $N \times N$ image. In contrast, *ghost imaging* enables image reconstruction from significantly fewer measurements by illuminating the object (x) with structured light patterns (S) and using a single-pixel detector to measure the total transmitted or reflected intensity[1,2].

The *sampling ratio (SR)* is defined as:

$$SR = \frac{M}{N^2}, \tag{1}$$

where K is the number of structured light patterns used. Each projected pattern ($S^{(m)}$) is modulated by the object and measured as a scalar intensity, commonly referred to as the bucket sum:

$$y^{(m)} = \sum_{i=1}^{N} \sum_{j=1}^{N} S_{i,j}^{(m)} \cdot x_{i,j}. \tag{2}$$

Early methods, such as Differential Ghost Imaging (DGI), used the bucket detector signals to compute weighted sums of the illumination patterns for object reconstruction, but these approaches often produced low-fidelity results [3]. More advanced methods recast ghost imaging as a linear system:

$$\mathbf{y} = \Psi \mathbf{x}, \tag{3}$$

where Ψ is the sensing matrix formed by flattening and stacking the illumination patterns, and $\mathbf{x}$ is the vectorized image. In this form, a standard solution using the Moore–Penrose Pseudoinverse (PI) is given by:

$$\mathbf{x} = \Psi^{\dagger} \mathbf{y}. \tag{4}$$

To enhance reconstruction quality, compressed sensing techniques leverage transform-domain sparsity[4–6], often employing ℓ_1 or ℓ_2 regularization, resulting in

$$\mathbf{y} = \Psi \Phi \boldsymbol{\alpha}, \quad \text{with } \mathbf{x} = \Phi \boldsymbol{\alpha}. \tag{5}$$

Here, Φ is a sparsifying basis (e.g., DCT or wavelets), and $\boldsymbol{\alpha}$ is a sparse coefficient vector. Solutions are obtained using iterative optimization methods such as Iterative Hard Thresholding (IHT)[4], Fast Iterative Shrinkage-Thresholding Algorithm (FISTA)[5], or Alternating Direction Method of Multipliers (ADMM)[6].

Deep neural networks and generative models have been used to improve image reconstruction fidelity[7]. One common approach first applies a classical reconstruction algorithm to produce a low-quality image, which is then enhanced using a trained neural network—such as a CNN, U-Net, or, more recently, a diffusion model[8–10]. This method benefits from the ability to incorporate learned priors for denoising and super-resolution. However, its performance depends on the quality as well as speed of the initial reconstruction and requires large datasets of paired examples. Another approach uses an end-to-end strategy by embedding elements of the ghost imaging process directly into the neural network architecture[11,12]. This method is typically faster, as it bypasses classical reconstruction algorithms entirely. However, prior studies have been constrained to low-resolution images (e.g., 28×28) and relied on binary mask patterns. Complementing these challenges on the algorithmic side, hardware constraints remain a significant bottleneck; however, dual optical frequency combs offer a promising solution.

Variable Definitions:

- $S^{(m)} \in \mathbb{R}^{N \times N}$: The mth structured illumination pattern,
- $x \in \mathbb{R}^{N \times N}$: The unknown image to be reconstructed,
- $y^{(m)}$: Total detected intensity for the mth pattern,
- $\mathbf{y} \in \mathbb{R}^{M \times 1}$: Vector of all scalar intensity measurements,
- $\Psi \in \mathbb{R}^{M \times N^2}$: Sensing matrix composed of flattened patterns,
- $\mathbf{x} \in \mathbb{R}^{N^2 \times 1}$: Flattened version of the image x,

- $\Phi \in \mathbb{R}^{N^2 \times N^2}$: Sparsifying basis (e.g., DCT, wavelets),
- $\boldsymbol{\alpha} \in \mathbb{R}^{N^2 \times 1}$: Sparse coefficients in the transform domain.

II. EXPERIMENTAL DETAILS

A. Dual Electro-Optic Comb Source

A continuous-wave (CW) fiber laser at 1550 nm is amplified and split into two paths using a 50/50 fiber coupler. Before splitting, a Fiber Bragg Grating (FBG) filter suppresses amplified spontaneous emission (ASE) noise. Each path is polarization-controlled and frequency-shifted via an acousto-optic frequency shifter, introducing a center frequency offset Δf_{center}. The beams are then modulated by resonant EO modulators (OptoComb WTEC-02-25) driven at f_{FSR} and $f_{\text{FSR}} + \Delta f_{\text{FSR}}$, where typical parameters are:

$$f_{\text{FSR}} = 20\,\text{GHz},\ \Delta f_{\text{FSR}} = 200\,\text{Hz} \quad \text{(static imaging)},$$

$$f_{\text{FSR}} = 25\,\text{GHz},\ \Delta f_{\text{FSR}} = 3\,\text{kHz} \quad \text{(dynamic imaging)}.$$

The resulting dual EO combs are recombined via a second 50/50 fiber coupler and re-amplified to offset insertion losses.

B. Free-Space Imaging Setup

A programmable Waveshaper (Coherent WS-01000B) is positioned before the free-space system to optionally filter individual comb lines. For bucket-sum measurements, the full dual-comb spectrum is transmitted. The output is coupled into a 5-meter multimode fiber (MMF) supporting hundreds of spatial modes, producing distinct speckle patterns when outcoupled into free space. These speckles are collimated and projected onto a transmissive negative USAF 1951 resolution target mounted on a motorized translation stage.

A 50/50 beam splitter separates the beam into signal and reference arms prior to the target, enabling simultaneous acquisition of interferograms and suppression of common-mode noise. Bucket measurements are recorded using InGaAs photodetectors (Thorlabs PDA50B2, 510 kHz bandwidth, 5 mm diameter active area). Since the actual focused beam spot has a diameter smaller than 1 mm, photodetectors with a smaller active area and higher bandwidth could be used in principle, especially when detecting a large number of comb lines or operating with a larger Δf_{FSR}. While the current reference arm is free-space, a fiber-based version using a 50/50 coupler is feasible and may be more practical. Reflection-mode imaging with a detector-integrated fiber front-end probe is also possible as an alternative to the current transmission configuration.

C. Speckle Basis Acquisition

The speckle basis set (H) without the target is recorded using a 2D InGaAs camera (Pembroke Instruments SenS-320V-ST) by selecting individual comb lines via the Waveshaper, either before or after imaging. The MMF was fixed to the optical table, ensuring stable speckle patterns over several hours and supporting repeatable experiments. In different imaging experiments, 150–200 comb lines were used in the optical C-band.

To improve the robustness of generated speckle patterns, one possible approach is to use an all-single-mode fiber design, where the SMF tip is coated with either a rough scattering layer or multi-layered metasurfaces to create a compact wavelength-dependent speckle/structured-light generator[13,14]. Such a method could minimize the effects of environmental variations and improve the stability and reliability of the generated speckle patterns.

D. Spectral Correlation Linewidth Characterization

To quantify the frequency-dependent stability of MMF-generated speckle patterns, we characterized the spectral correlation linewidth using an independent tunable CW laser (not the comb source). The laser was tuned from 1530 to 1580 nm in 10 nm steps, and finely scanned around each central wavelength while output speckle images were

recorded. The Pearson correlation between central and detuned speckle images was computed, and the full width at half maximum (FWHM) of the correlation function defined the spectral correlation linewidth.

Measurements were performed for two fibers: a 5-m MMF with 105 μm core diameter (Fig. S2A) and a 50-m MMF with 105 μm core diameter (Fig. S2B). As summarized in Fig. S2C, the 5-m fiber exhibited a linewidth of 22.1 $\pm$ 2.2 GHz, whereas the 50-m fiber yielded a narrower linewidth of 1.4 $\pm$ 0.1 GHz. The reduction in linewidth with fiber length arises from stronger modal dispersion and provides finer wavelength sensitivity. Such narrow correlation linewidths are advantageous for future implementations, enabling the use of smaller comb FSRs to increase the sampling ratio in hyperspectral dual-comb compressive imaging.

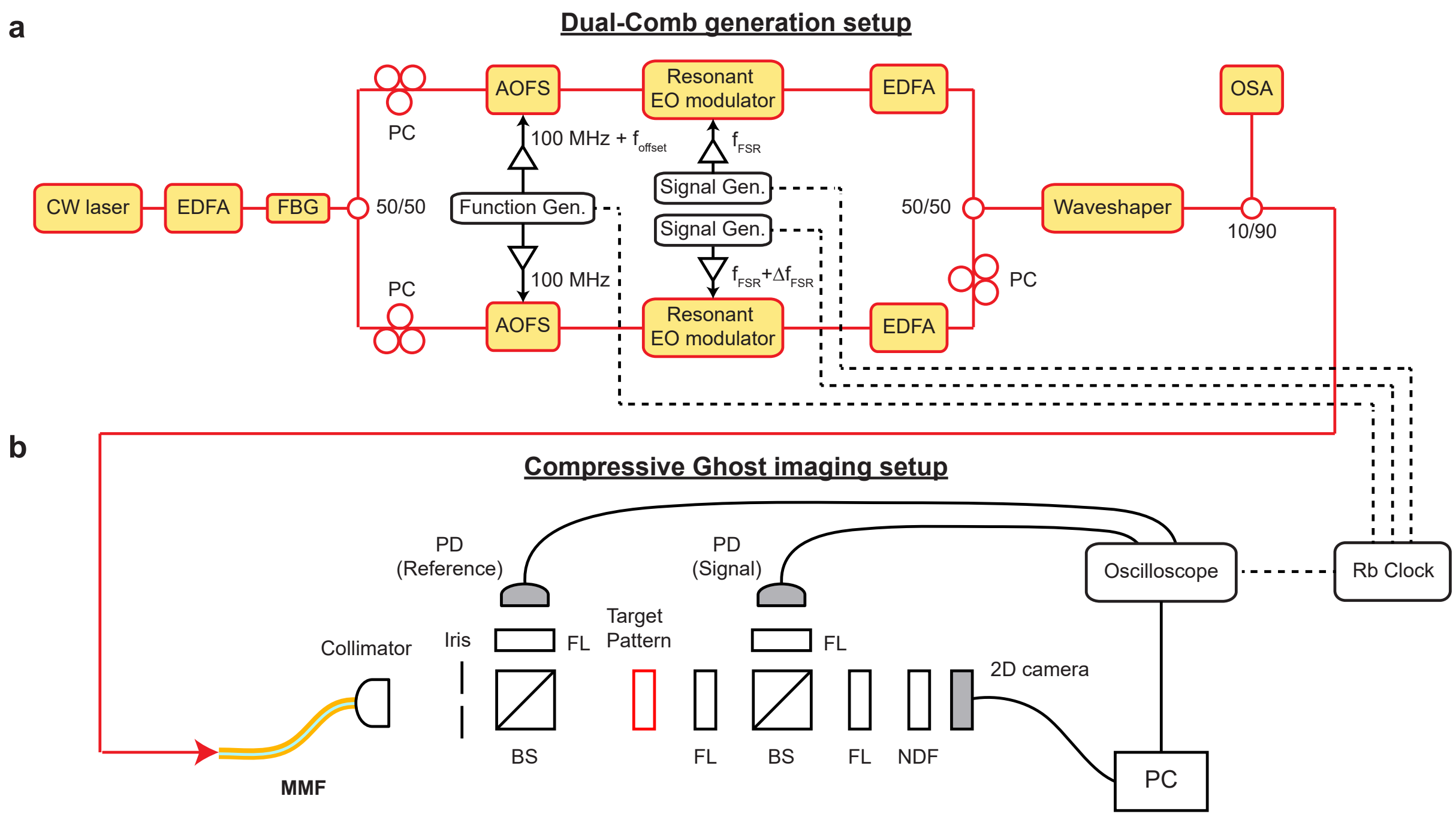

Fig. S1: Experimental Setup. a, Dual-Comb generation setup. **b**, Free-space compressive Ghost imaging setup. For improved SNR, a function generator, two signal generators, and an oscilloscope were referenced to an external timing reference (Rb clock; Stanford Research Systems FS740). EDFA: Erbium Doped Fiber Amplifier (Thorlabs EDFA300P), FBG: Fiber Bragg Grating (Dicon TF2-1550-0.6-9/9LT-FC/APC-H-1), AOFS: Acousto-Optic Frequency Shifter (Brimrose TEM-100-18-30-1550-2FP-HP-PM), SMF: Single Mode Fiber, MMF: Multi Mode Fiber, OSA: Optical Spectrum Analyzer, PD: Photodetector (Thorlabs PDA50B2), FL: Focusing Lens (Thorlabs AC254 Ø1" Achromatic Doublets), BS: Beam Splitter (Thorlabs BS015 50:50 Non-Polarizing Beamsplitter Cube, 1100 - 1600 nm, 1"), NDF: Neutral Density Filter.

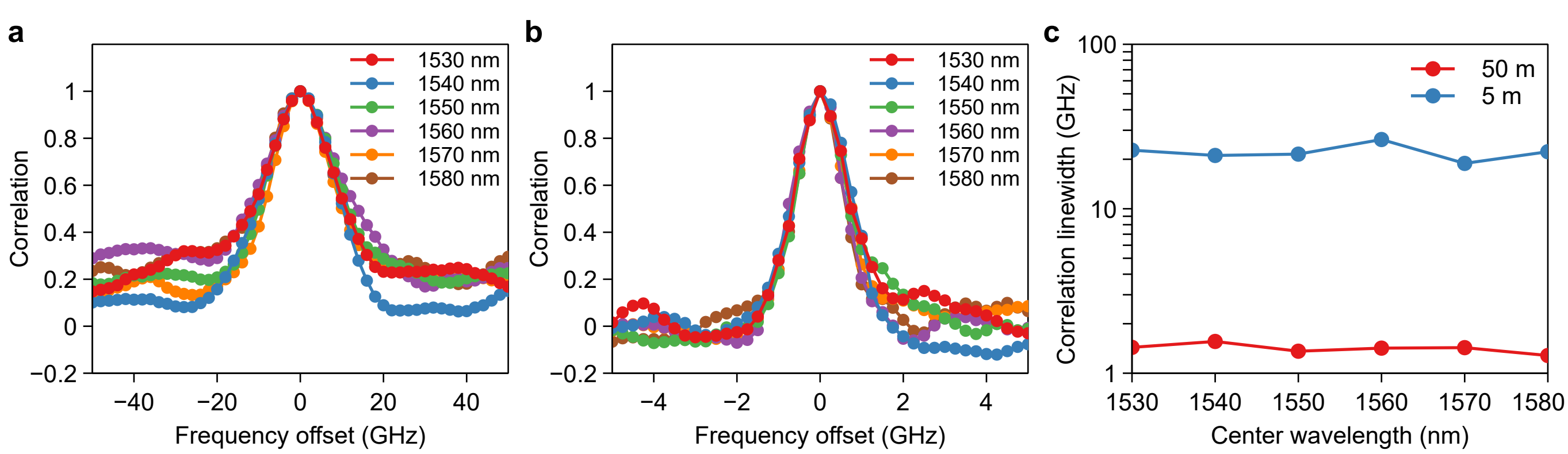

Fig. S2: Spectral correlation linewidth of MMF-generated speckle patterns. Spectral correlation curves measured for **a**, a 5-m MMF (105-µm core) and **b**, Same measurement for a 50-m MMF (105-µm core) at different wavelengths (1530–1580 nm). **c**, Extracted FWHM linewidths: 22.1 $\pm$ 2.2 GHz (5-m MMF) and 1.4 $\pm$ 0.1 GHz (50-m MMF).

III. GHOST-GPT: TRANSFORMER-BASED IMAGE RECONSTRUCTION MODEL

Transformers are a powerful deep learning architecture originally introduced for natural language processing (NLP) tasks but have since found applications in various domains[15], including computer vision[16], speech processing[17], and scientific data analysis[18]. They leverage a mechanism called self-attention to model long-range dependencies and capture contextual relationships within sequences[19]. Unlike traditional recurrent or convolutional networks, transformers process entire sequences simultaneously, making them highly efficient for parallel computation. Their ability to learn complex patterns from large datasets has made them the backbone of state-of-the-art models like Bidirectional Encoder Representations from Transformers (BERT), Generative Pre-trained Transformer (GPT)[20], and Vision Transformers (ViTs)[21][22].

In typical ViTs, the image is broken up into equally sized patches, which serve as the ViTs' token. In order to adapt this methodology to dual-comb ghost imaging, we propose concatenating the flattened illumination patterns that make the sensing matrix, Ψ, with the bucket value to form the token for our model.

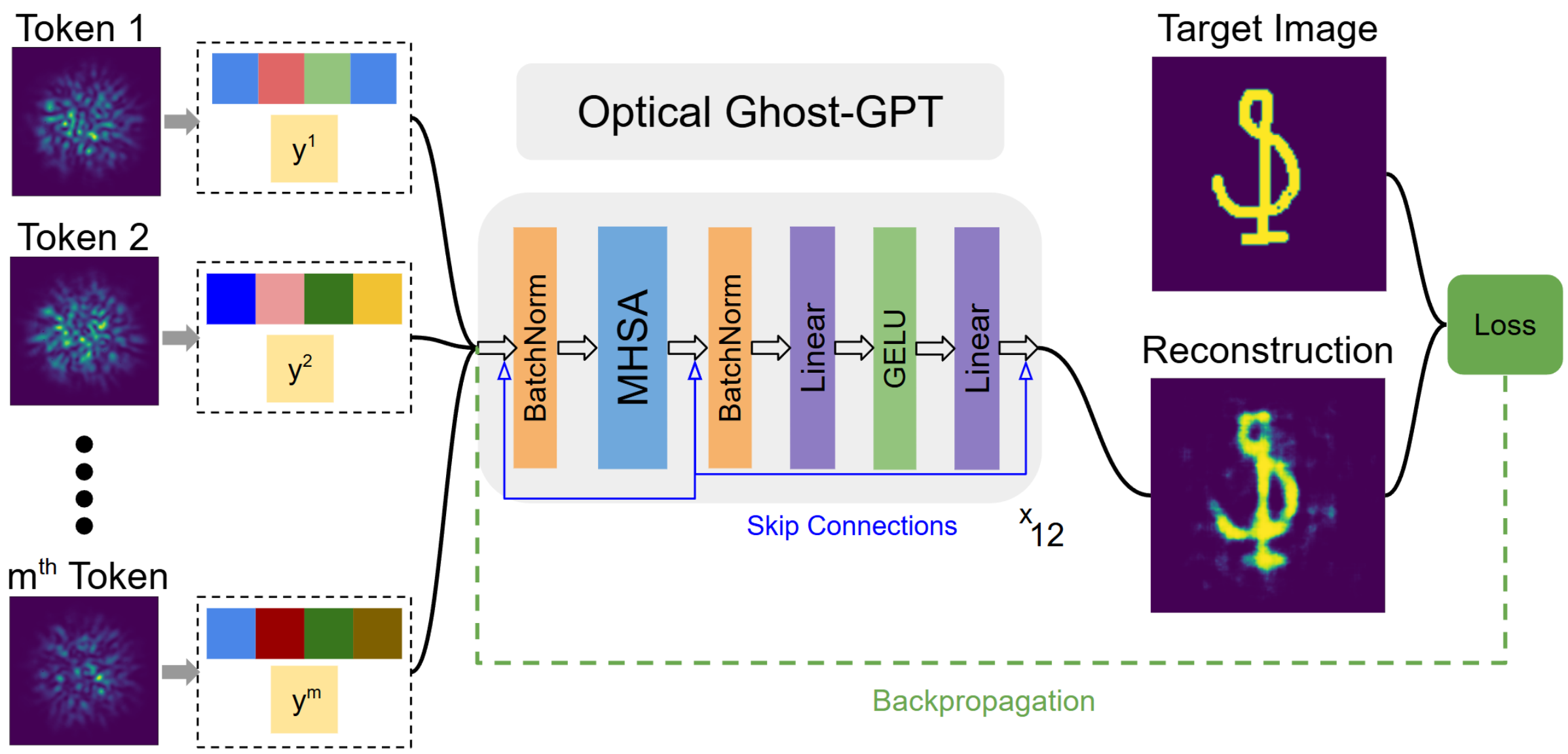

Fig. S3: Schematic of Optical Ghost-GPT. The speckle patterns are first compressed into a latent space and then concatenated with the corresponding bucket measurements to form the input token sequence.

A. Model Architecture.

We introduce **Optical Ghost-GPT** (or simply **Ghost-GPT**), a transformer-based model designed for structured image reconstruction from ghost imaging measurements. The model leverages stacked self-attention mechanisms and residual feedforward blocks to model long-range dependencies across the contextual input.

Input Embedding. The model receives two primary inputs in each token: a flattened image of the speckle pattern $\mathbf{\Psi}^m \in \mathbb{R}^N$, where $N = 256 \times 256$, and bucket sum value $\mathbf{y^m}$. The image vector is projected via a learnable linear transformation to a latent embedding of dimension $d_k - 1$, and afterwards, the latent representation of the image vector and its corresponding bucket sum are concatenated. To encode positional structure, we add learned positional embeddings to each token in the sequence:

$$\mathbf{z}_i = \mathbf{e}_i + \mathbf{p}_i, \quad i = 1, \ldots, C,$$

where $\mathbf{e}_i$ is the input token embedding, $\mathbf{p}_i$ is the corresponding positional embedding, and C=250 is the context size determined by the maximum number of RF comblines in our current setup.

Transformer Blocks. The architecture contains a stack of $L = 12$ transformer blocks, each composed of a multi-head self-attention (MHSA) mechanism and a two-layer feedforward network. The attention mechanism employs H number of heads, computed as:

$$\text{Attention}(Q, K, V) = \text{Softmax}\left(\frac{QK^\top}{\sqrt{d_k}}\right)V,$$

where Q, K, and V are linear projections of the input sequence, and d_k is the size of the latent space embedding. We also apply a dropout mask with a value of 0.1 to ensure robustness of the model and allow the model to generalize to missing shots and buckets. Each block includes residual connections and a batch normalization operation placed before and after the MHSA layer, defined as:

$$\text{BatchNorm}(\mathbf{x}) = \frac{\mathbf{x} - \mu}{\sqrt{\sigma^2 + \varepsilon}}, \quad \mu = \mathbb{E}[\mathbf{x}], \quad \sigma^2 = \text{Var}[\mathbf{x}].$$

The feedforward network consists of two feed-forward layers with a gaussian error linear unit (GELU) activation; A final transformer block is appended after the main stack to further refine the sequence representation.

$$\text{FFN}(\mathbf{x}) = \text{Linear}_2\left(\text{GELU}(\text{Linear}_1(\mathbf{x}))\right).$$

Output Projection. The final token representations are projected to $\mathbb{R}^{16}$ via a linear layer and the output is flattened to a tensor of size $(C \times 16)$ and passed through a final linear layer to reconstruct the original image vector in $\mathbb{R}^{256\times256}$. A sigmoid activation is applied to constrain the output to the range $[0, 1]$, consistent with normalized image intensities:

$$\hat{\mathbf{x}} = \sigma\left(\text{Linear}_{\text{final}}(\text{Flatten}(\mathbf{z}))\right).$$

Network Training. We first obtain a set of speckle patterns from the 2-D camera during the calibration phase of the experiment. (We emphasize that the 2-D camera is only needed to obtain the initial speckle patterns and can be removed during imaging). For this experiment, we obtained 188 speckle patterns, which are then used to generate synthetic buckets sums via a convolution between the digitized speckle pattern and images from the MNIST and OMNIglot datasets. We form our labeled dataset of synthetic bucket sums as the x-label and its corresponding target images as the y-label (See Section III D for dataset generation, train/test split). In training, Ghost-GPT predicts the target image, given the speckle pattern and bucket sums as the input. We use mean squared error as our loss function and an AdamW optimizer with a learning rate of 0.0003 and a weight decay of 0.1.

B. Ghost-GPT Reconstruction Results

In this section, we examine the reconstruction results in simulation using our experimental speckle pattern results. Figure S4 shows a series of reconstruction compared with their true images.

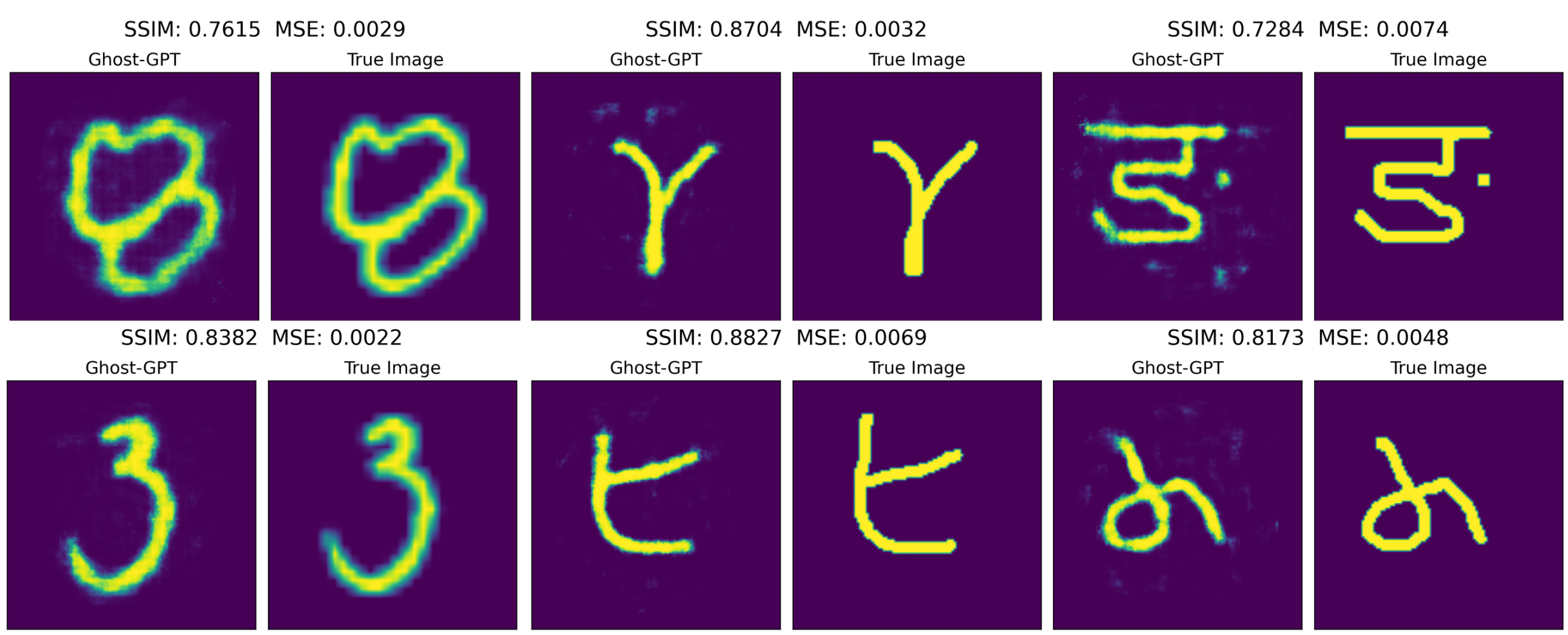

Fig. S4: Simulated ghost imaging reconstructions from Ghost-GPT. Reconstructions from Ghost-GPT versus the true image with the Structural Similarity Index Measure (SSIM) and Mean Squared Error (MSE) displayed above.

We emphasize that we achieve very high SSIM (greater than 0.7) and low MSE (less than 0.02) values despite having a sampling ratio of only 0.29%. We observe that the model is able to preserve fine structural details and clear object boundaries, highlighting the model's ability to recover meaningful image content from minimal measurements. However, the reconstructions can exhibit variations in intensity across the object, especially in images with long, thin

structures or uniform intensity profiles. These artifacts likely stem from the inherent non-uniformity of the speckle patterns generated by the multi-mode fiber. This issue could potentially be mitigated by employing a more uniform speckle distribution or by including a smoothness term, such as total variational loss, in the loss function.

We also compared our model against classical reconstruction algorithms on 256 images from our validation dataset. (256 images were chosen due to the long reconstruction times associated with the iterative FISTA algorithm). Table S1 compares the MSE and SSIM of previously discussed reconstruction algorithms- as expected the simpler reconstruction algorithms such as Differential Ghost Imaging and the Moore-Penrose Psuedo Inverse perform worse than compressed sensing methods like FISTA. However, Ghost-GPT outperforms these classical algorithms giving an average **MSE of 0.008** and **SSIM of 0.788**, while being approximately **263x** faster than FISTA. (We set $\epsilon = 50$ after performing a hyperparameter sweep and choosing the best performing SSIM). The extremely fast reconstruction speed of **14 ms** enables **real-time, video frame-rate ghost imaging in optical fibers**. Importantly, while the computational reconstruction speed can be further improved with better computing hardware, the fundamental limit of image reconstruction is set by the repetition rate difference of the dual-comb, which is typically a few hundred Hz to several kHz in our experiments. With a larger repetition rate difference, a much higher frame rate is possible. However, this requires high-bandwidth photodetection, and the trade-off between the sampling ratio and frame rate must be considered in accordance with the Nyquist condition.

TABLE S1: Classical Algorithms vs Ghost-GPT

Algorithm	MSE	SSIM	Computational Time (ms)
Differential Ghost Imaging	0.184 (σ=0.037)	0.042 (σ=0.022)	15.7 (σ=0.138) GPU
Moore–Penrose Pseudo-Inverse	0.093 (σ=0.027)	0.055 (σ=0.027)	5640 (σ=52.1) CPU<br>157 (σ=3.27) GPU
FISTA (200 iters, ϵ=50)	0.045 (σ=0.017)	0.092 (σ=0.019)	3680 (σ=125) CPU<br>10500 (σ=93.8) GPU
**Ghost-GPT (Ours)**	**0.008 (σ=0.009)**	**0.788 (σ=0.076)**	**14.0 (σ=0.457) GPU**

C. Robustness of Ghost-GPT in Experimental Imaging

To examine the effect of noise in our experimental measurement, we performed a signal-to-noise ratio analysis by adding varying amounts of noise to simulated buckets and gauged the quality of image reconstructions (See Fig. S5). The artificial noise added to the buckets were calculated by sampling points from a gaussian with mean of zero and standard deviation of one and scaling it inversely proportional to the value of the SNR (See Section III F for more information about the SNR calculation). Ghost-GPT demonstrates strong robustness to noise, outperforming classical reconstruction algorithms, with recognizable reconstructions achievable at SNR levels above 20 dB for Ghost-GPT. (See Fig. S7 for plots of the calculated MSE and SSIM values vs SNR). Based on the image reconstruction quality of both the classical reconstruction algorithm and Ghost-GPT in the SNR analysis, our experimental bucket measurements are approximately equivalent to 25-30 dB.

D. Dataset Generation, Train and Test Split

Since the bucket signal is effectively a convolution between the object and each speckle pattern, it is computationally efficient to synthetically generate labeled training data by pairing known objects with their corresponding bucket sums. For this, we use open-source image datasets and simulate the measurement process. Our dataset includes 19,280 from OMNIglot and 19,280 images from MNIST with a train/val split of 33,000/5,560 respectively. All images are resized to 256 $\times$ 256 to match the resolution of the recorded speckle patterns. We use a batch size of 32 for the training dataset and a batch size of 64 for the validation dataset.

E. Hyperparameter Analysis

For hyperparameter tuning, we performed a simple parameter sweep by changing the number of attention heads at values of 8, 16, 24, and 32 as well as adjusting the embedding dimension at values of 1 (no pattern embedding), 8, 16, 32, and 64. As previously stated, we use mean squared error as our loss function and an AdamW optimizer with a learning rate of 0.0003 and a weight decay of 0.1. In our loss function, we add the error in each pixel and then average over the batch size.

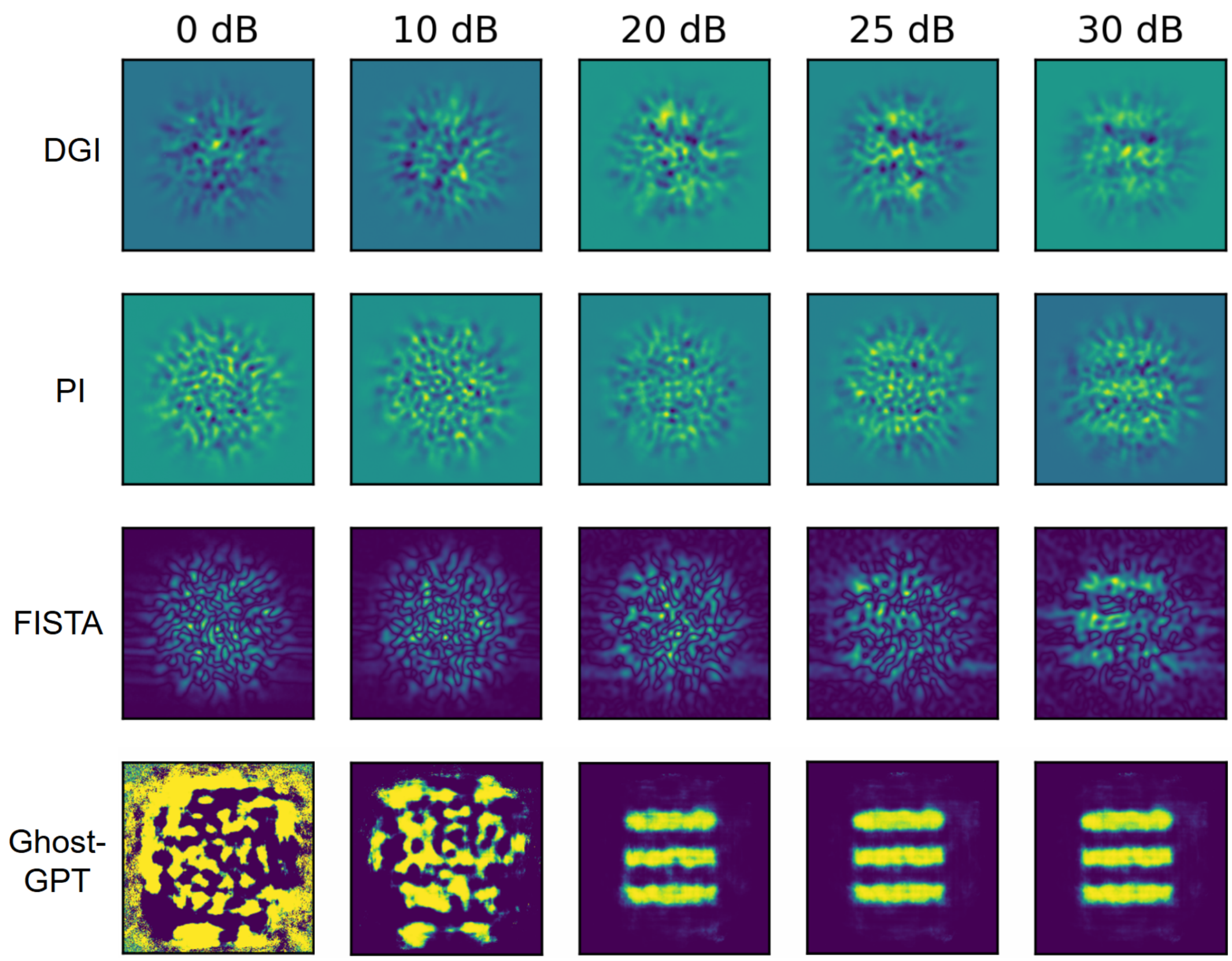

Fig. S5: Ghost-GPT performance across various signal-to-noise ratios. Simulated reconstructions of the USAF striped targets with artificial noise added to the buckets. The SNR in dB is shown label at the top with the corresponding reconstruction algorithms on the left-hand side.

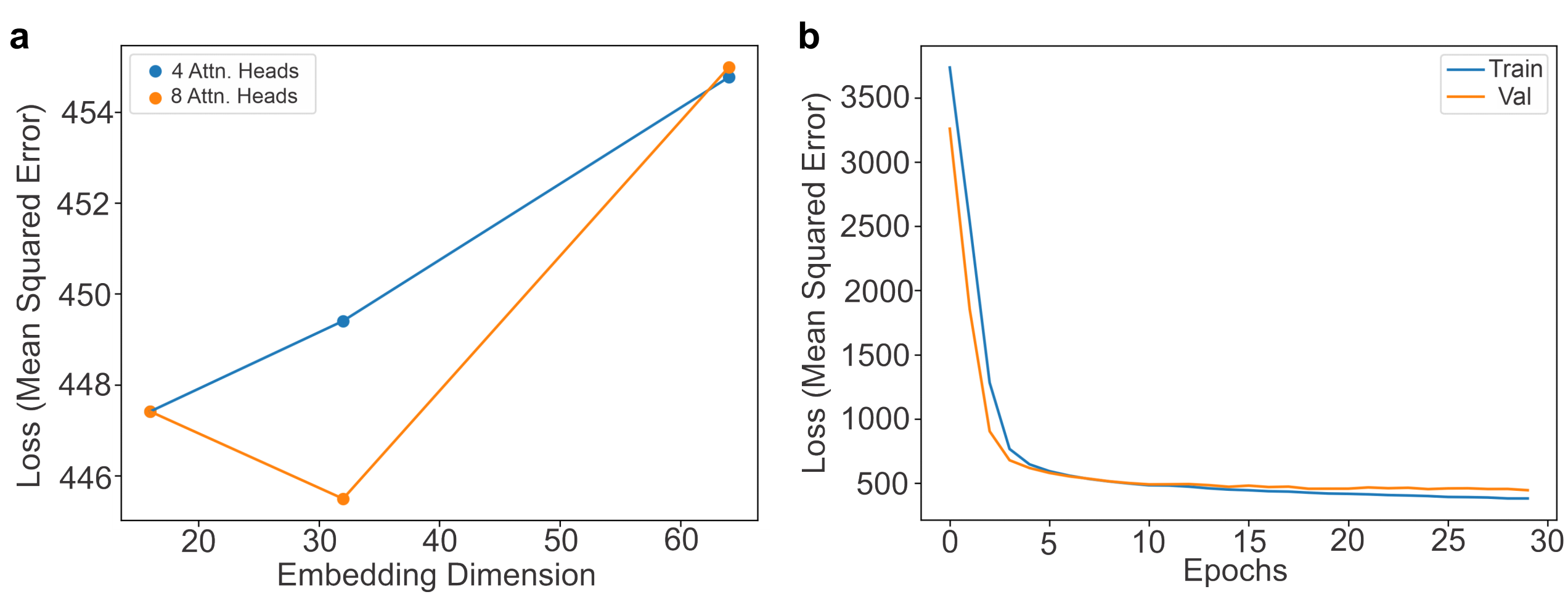

Fig. S6: Hyperparameter tuning of attention heads and embedding dimensions. a, The lowest loss value on the validation dataset as a function of the number of attention heads and embedding dimensions. **b**, Loss history of the model with 32 attention heads and an embedding dimension of 64.

Over 50 epochs, the model took approximately 2 hours to train on a Linux workstation, using an AMD Ryzen Threadripper 3990X 64-Core Processor and a single NVIDIA A6000 GPU. Fig. S6a shows that Ghost-GPT generally performs better as we increase the size of the model - indicating that with larger embedding dimensions the model is able to understand and model short/long range dependencies between the speckle patterns more effectively. Our largest

model (32 attention heads, embedding dimension 64) contained $\sim$ 269 million trainable parameters and demonstrates convergence afters 10 epochs (see Fig. S6b) - displaying no characteristics of overfitting.

F. Different Algorithms vs Signal-to-Noise Ratio

The signal-to-noise ratio (SNR) was calculated via the following equation:

$$\mathbf{y}_{noisy} = \mathcal{N}(0,1) * \sigma + \mathbf{y}, \quad \sigma = \frac{\mathbb{E}[\mathbf{y}]}{\text{SNR}^{10}}$$

We sample points from a gaussian of mean 0 and std 1, which is scaled by σ, the standard deviation. σ is calculated the by averaging over the collection of bucket sum and then dividing by the SNR value raised to the 10th power. The true bucket sum, $\mathbf{y}$, is added to the previously calculated quantity to create the noisy bucket, $\mathbf{y}_{\text{noisy}}$. Previous works in ghost imaging formulated their SNR analysis using these exact definitions.

Fig. S7 plots the MSE and SSIM of the reconstructed images compared with classical reconstruction algorithms and Ghost-GPT.

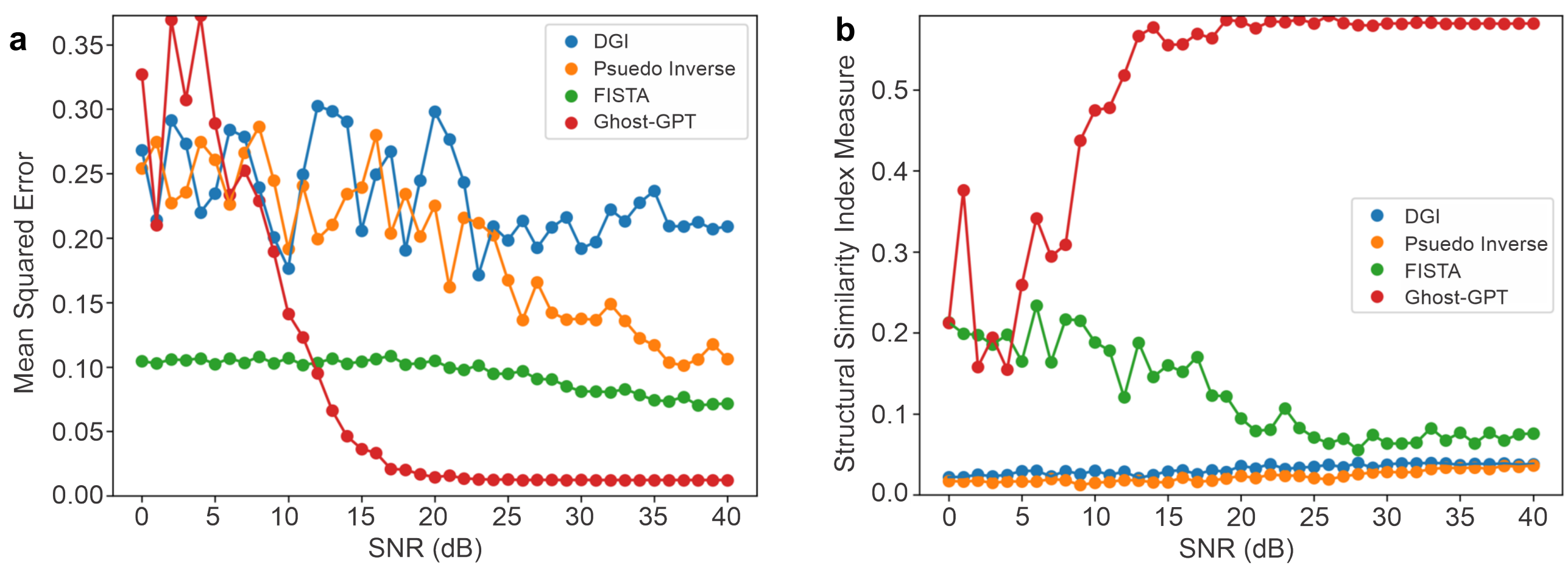

Fig. S7: Reconstruction quality of various algorithms as a function of SNR. Artificial noise is added to the digtal bucket sum and their reconstruction quality is quantified by **a**, MSE and **b**, SSIM of the USAF target.

G. Resolution Analysis

In ghost imaging, resolution is fundamentally linked to the spatial characteristics of the light patterns used to probe the object—specifically, the grain size of these patterns. Grain size refers to the typical scale or correlation length of the intensity fluctuations in the illumination patterns (often speckle patterns). This grain size determines the finest detail that can be distinguished in the reconstructed image.

In this section, we evaluated the smallest resolvable feature size achievable by Ghost-GPT in simulation using our current experimental speckle pattern set (See Fig. S8. A digital resolution target was generated with 15-pixel-wide bars and varying gap sizes (1, 3, 5, 7, 9 pixels). Ghost-GPT successfully resolved features with a 5-pixel gap. In our experimental setup, the Air Force resolution target's 23-pixel feature between a pair of stripes corresponds to a physical size of 0.355 mm, indicating that Ghost-GPT achieves a resolution of approximately 0.077 mm. At this resolution, our ghost imaging model is well-suited for visualizing a broad range of biological structures, including tissue architecture such as blood vessels, skin layers, and muscle fibers. It also enables observation of developmental forms in small organisms like embryos and larvae, as well as structural features in plant tissues such as roots and leaves.

H. Comparison with Other Deep Learning Models

For the image reconstruction task, we choose transformer architecture, since naturally, the self-attention mechanism seemed well suited for dealing with the image speckle patterns and image bucket sums. To further validate our choice

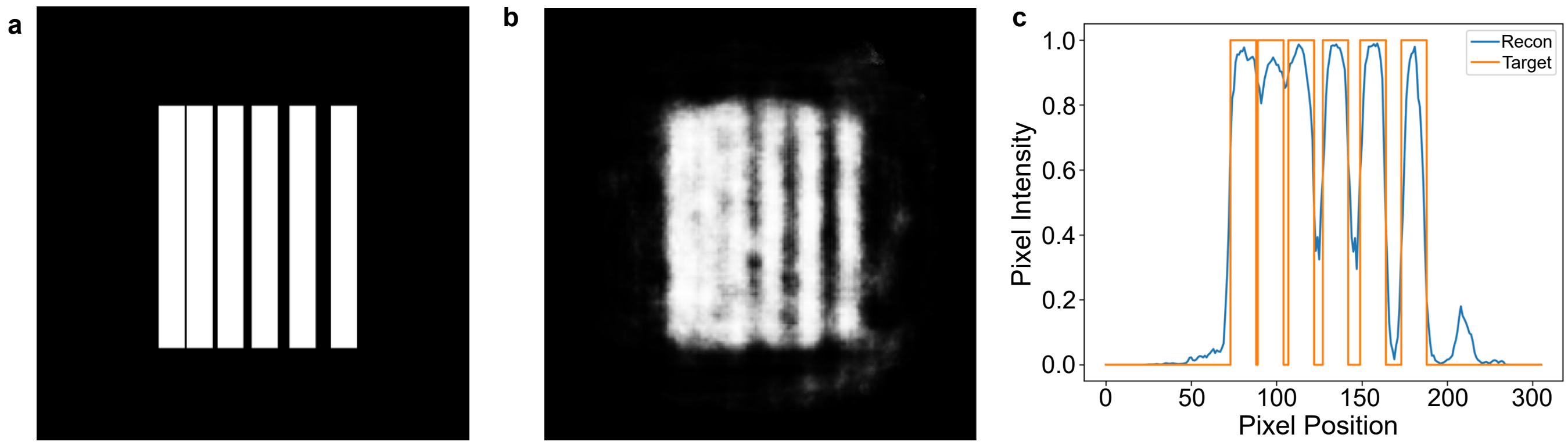

Fig. S8: Simulated resolution testing of Ghost-GPT. **a**, True resolution target with increasing pixel gaps. **b**, Ghost-GPT reconstruction results. **c**, Line profile comparing the ground truth and model output, obtained by scanning along a horizontal cross-section midway through the images.

of model architecture, we compared our Ghost-GPT model with a convolutional neural network (CNN) and U-Net. We used approximately the same number of parameters (∼270 million parameters for both CNN and U-Net) and also used the same bucket sum and speckle pattern concatenation scheme as the input to these models.

TABLE S2: Image Reconstruction Quality on Various Neural Network Architectures

Model Architecture	MSE	SSIM	Computational Time (ms)
**Ghost-GPT (Ours)**	**0.0068 (σ=0.0077)**	0.787 (σ=0.073)	14.0(σ=0.457) (GPU)
CNN	0.013 (σ=0.0086)	0.8079 (σ=0.0395)	**6.26 (σ=0.41) (GPU)**
UNet	0.011 (σ=0.0073)	**0.8316 (σ=0.0696)**	15.09 (σ=0.032) (GPU)

The CNN baseline is implemented as a decoder-only architecture that first projects the input measurement vector through a large fully connected layer (Linear(M → C·H/8·W/8)), reshaping it into a coarse feature map, followed by upsampling through three stages of transposed convolutions. Batch normalization and ReLU are applied after the first two deconvolutional layers, and the output is produced with a final transpose convolution to a single channel. In contrast, the U-Net employs a full encoder–decoder structure with convolutional blocks at multiple scales. The encoder uses repeated double 3×3 convolutions and max-pooling for downsampling, while the decoder mirrors this with transposed convolution upsampling, skip connections from the encoder, and additional double convolutions at each stage. Batch normalization is applied consistently across all blocks, and the network terminates with a 1×1 convolutional head. Parameter distribution also differs: in the CNN, the majority of weights reside in the large fully connected input layer, whereas in the U-Net they are concentrated in the deeper convolutional bottleneck and wide decoder stages.

In Table S2, Ghost-GPT achieves a lower mean-squared error (MSE) than both CNN (46.2% reduction) and U-Net (36.4% reduction). Note that this table examines MSE and SSIM over the entire validation dataset instead of a small sample of it. Since our model is trained with MSE as the loss function, this metric most directly reflects its reconstruction performance. At the same time, SSIM results indicate that Ghost-GPT performs slightly better than CNN but somewhat lower than U-Net, highlighting that its gains in MSE are balanced by broadly comparable structural similarity. In terms of computational efficiency, Ghost-GPT is the second fastest among the baselines, offering a favorable tradeoff between accuracy and speed. Figure S9 further shows that our model converges to a lower loss within the same number of epochs compared to the other architectures. Overall, these results suggest that Ghost-GPT's primary strength lies in minimizing MSE, while maintaining competitive performance across perceptual quality and runtime.

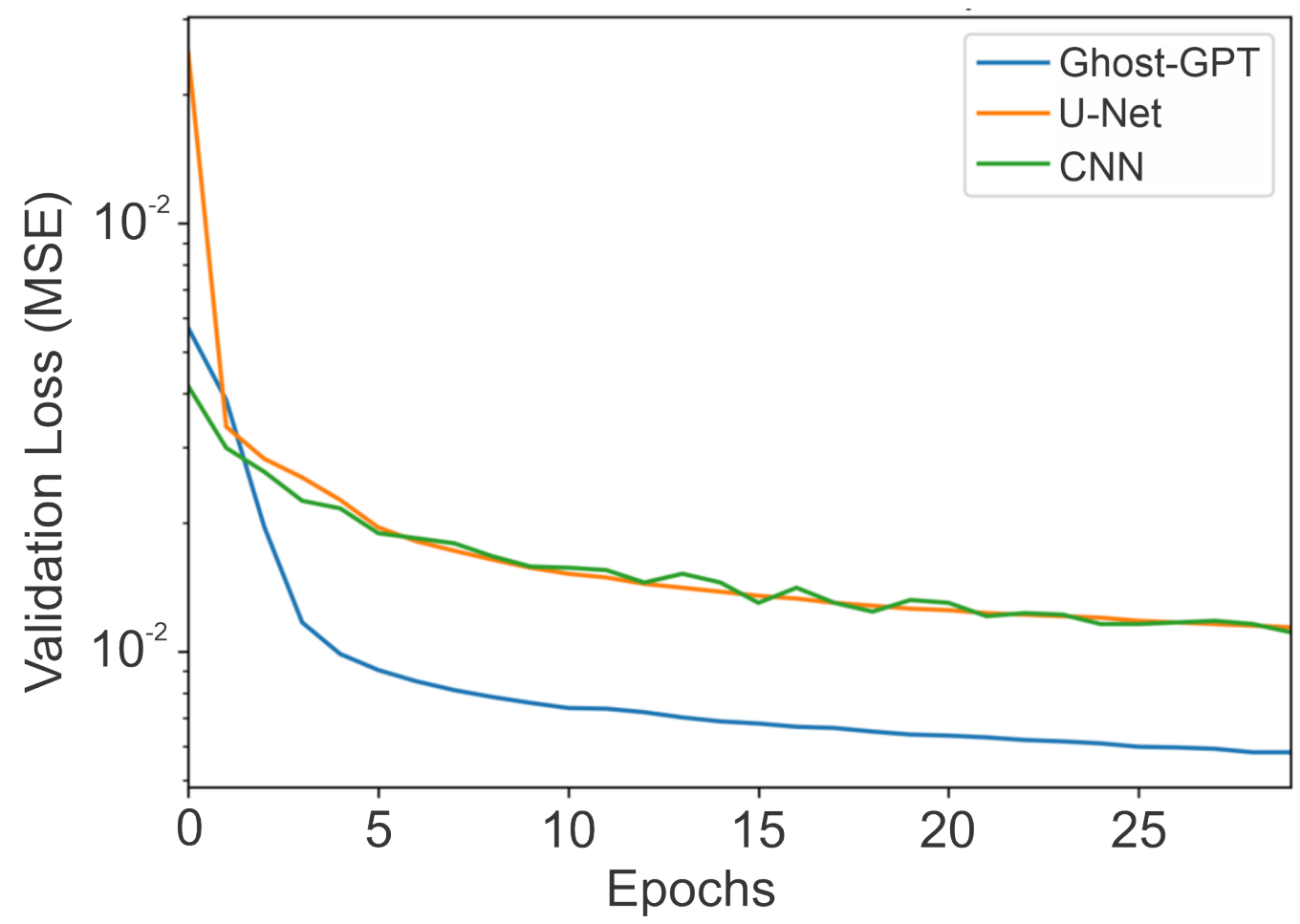

Fig. S9: Loss history of different deep learning models. Our GPT model outperforms both CNN and UNet consistently at almost all epochs in terms of MSE.

I. Experimental Hyperparameter Tuning

Due to the effects of noise in the experimental data, the reconstructed images have visual artifacts and degraded image quality, such as the presence of rough edges and artificial spots. As a result, we tuned our model further by adjusting the mean squared error (MSE) loss function to also include a total variational (TV) term to help improve the experimental image quality. The resulting equation is defined as:

$$Loss = MSE + \beta * TV \tag{6}$$

The addition of total variational loss in the model helps to smooth artifacts in the image at the cost of overall image sharpness. We weighed the TV by a factor, β, and performed a hyperparameter sweep between 0.1 and 3 to find the right balance between the two terms in the loss function (Fig. S10 and Table S3). The values of β was determined by evaluating our model on our experimentally measured ghost imaging targets and choosing β with the highest SSIM reconstructions. We found that a β of 1.5 was the ideal value; this corresponding model and its reconstruction results are shown in the main manuscript.

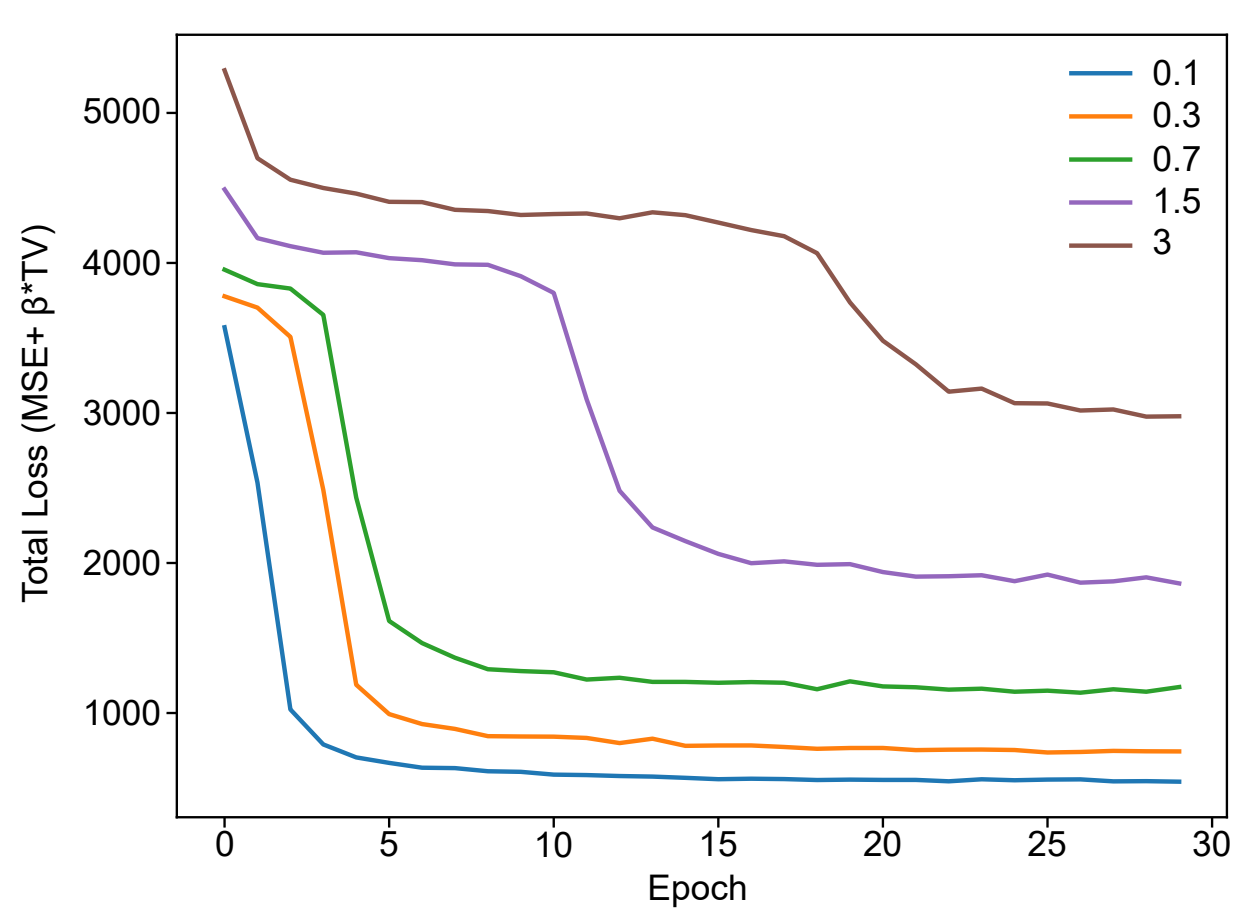

Fig. S10: Total variational loss tuning. Loss history with the addition of the total variational loss term, which is weighted by a scalar value β. The value of β is shown by the different colors within the plot. Convergence of the network occurs within 50 epochs.

TABLE S3: Average SSIM on Noisy Validation Dataset

TV weight (β)	0.1	0.3	0.7	1.5	3
Stripes (SSIM)	0.493	0.539	0.539	**0.560**	0.351
Number 2 (SSIM)	0.558	0.608	0.633	**0.634**	0.482

J. Ghost Imaging of Natural Images

In our work, we demonstrated ghost imaging of binary targets due to the limited number of available comblines. This restricts our sampling ratio to ∼0.3%, which is too small properly resolve details within a intricate, grayscale image. Imaging more complex, natural objects becomes feasible when the number of speckle patterns or comblines is increased to achieve a sufficiently high sampling ratio[8,23]. The lower limit of the sampling ratio greatly depends on the imaging reconstruction algorithm and desired image quality, but sampling ratios as low as ∼ 1% have been used to generate recognizable natural images[24].

To demonstrate that ghost imaging of natural scenes is achievable with Ghost-GPT, we trained our model on a dataset of 64×64 images of dogs and used random noise patterns as simulated uncorrelated speckle patterns. We tested image reconstruction with 256, 512, 1024, and 2048 illumination patterns using Ghost-GPT, corresponding to sampling ratios of 6.25%, 12.5%, 25%, and 50%, respectively. As shown in Fig. S11, the complex features of the target images become increasingly detailed as the sampling ratio increases, with only minor improvements in image quality beyond a 25% sampling ratio. With an increased number of comb lines (and a correspondingly larger sampling ratio), this demonstrates that our ghost imaging approach with Ghost-GPT can be generalized to more practical environments and scenes, such as the imaging of human tissue.

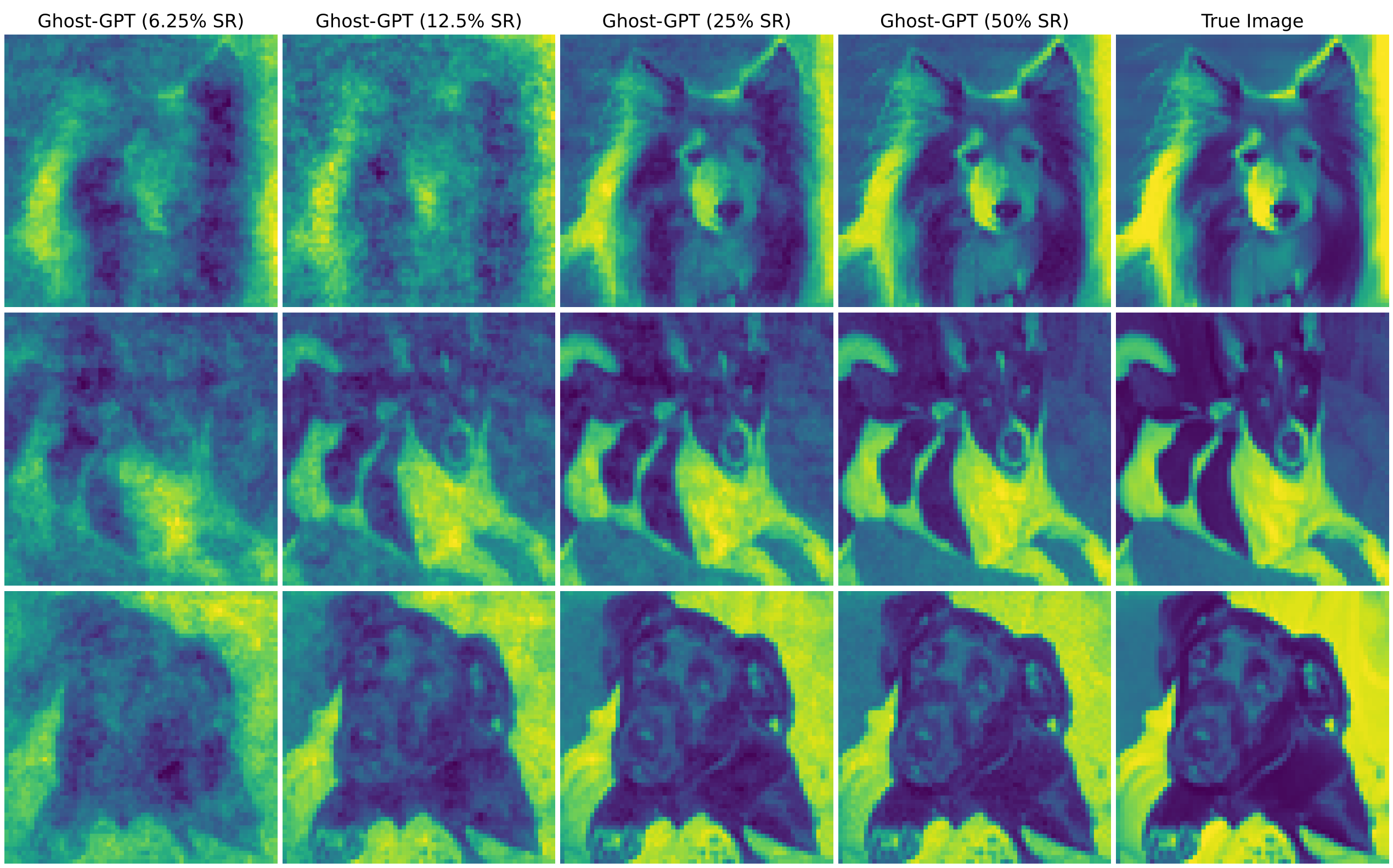

Fig. S11: Natural Imaging with Ghost-GPT Example reconstructed images with Ghost-GPT using different sampling ratios.

[1] Li, S. *et al.* Electron ghost imaging. *Physical review letters* **121**, 114801 (2018).
[2] Bromberg, Y., Katz, O. & Silberberg, Y. Ghost imaging with a single detector. *Phys. Rev. A* **79**, 053840 (2009). URL `https://link.aps.org/doi/10.1103/PhysRevA.79.053840`.
[3] Liu, H.-C. Imaging reconstruction comparison of different ghost imaging algorithms. *Scientific Reports* **10**, 14626 (2020).
[4] Blumensath, T. & Davies, M. E. Iterative hard thresholding for compressed sensing. *Applied and computational harmonic analysis* **27**, 265–274 (2009).
[5] Beck, A. & Teboulle, M. A fast iterative shrinkage-thresholding algorithm for linear inverse problems. *SIAM journal on imaging sciences* **2**, 183–202 (2009).
[6] Boyd, S. *et al.* Distributed optimization and statistical learning via the alternating direction method of multipliers. *Foundations and Trends® in Machine learning* **3**, 1–122 (2011).
[7] Rizvi, S., Cao, J., Zhang, K. & Hao, Q. Deepghost: real-time computational ghost imaging via deep learning. *Scientific reports* **10**, 11400 (2020).
[8] He, Y. *et al.* Ghost imaging based on deep learning. *Scientific reports* **8**, 6469 (2018).
[9] Wang, F. *et al.* Far-field super-resolution ghost imaging with a deep neural network constraint. *Light: Science & Applications* **11**, 1 (2022).
[10] Mao, S. *et al.* High-quality and high-diversity conditionally generative ghost imaging based on denoising diffusion probabilistic model. *Optics Express* **31**, 25104–25116 (2023).
[11] Ren, W., Nie, X., Peng, T. & Scully, M. O. Ghost translation: an end-to-end ghost imaging approach based on the transformer network. *Optics Express* **30**, 47921–47932 (2022).
[12] Liang, J., Cheng, Y. & He, J. Transformer-based flexible sampling ratio compressed ghost imaging. *Engineering Analysis with Boundary Elements* **170**, 106050 (2025).
[13] Shin, J., Bosworth, B. T. & Foster, M. A. Single-pixel imaging using compressed sensing and wavelength-dependent scattering. *Optics letters* **41**, 886–889 (2016).
[14] Zhu, R. *et al.* Compact fiber-integrated scattering device based on mixed-phase tio2 for speckle spectrometer. *Optics Letters* **47**, 1606–1609 (2022).
[15] Rothman, D. *Transformers for Natural Language Processing: Build, train, and fine-tune deep neural network architectures for NLP with Python, Hugging Face, and OpenAI's GPT-3, ChatGPT, and GPT-4* (Packt Publishing Ltd, 2022).
[16] Jamil, S., Jalil Piran, M. & Kwon, O.-J. A comprehensive survey of transformers for computer vision. *Drones* **7**, 287 (2023).
[17] Kashyap, S., Singh, S. & Singh, D. V. Speech-to-speech translation using transformer neural network. In *International conference on soft computing for problem-solving*, 813–826 (Springer, 2023).
[18] Sufi, F. Generative pre-trained transformer (gpt) in research: A systematic review on data augmentation. *Information* **15**, 99 (2024).
[19] Vaswani, A. *et al.* Attention is all you need. *Advances in neural information processing systems* **30** (2017).
[20] Ghojogh, B. & Ghodsi, A. Attention Mechanism, Transformers, BERT, and GPT: Tutorial and Survey (2020). URL `https://hal.science/hal-04637647`. Working paper or preprint.
[21] Maurício, J., Domingues, I. & Bernardino, J. Comparing vision transformers and convolutional neural networks for image classification: A literature review. *Applied Sciences* **13**, 5521 (2023).
[22] Zhang, P. *et al.* Multi-scale vision longformer: A new vision transformer for high-resolution image encoding. In *Proceedings of the IEEE/CVF international conference on computer vision*, 2998–3008 (2021).
[23] Don, M. An introduction to computational ghost imaging with example code. *CCDC Army Research Laboratory: Aberdeen Proving Ground, MD, USA* (2019).
[24] Chen, Y. *et al.* Large model enhanced computational ghost imaging. *Science China Technological Sciences* **68**, 2120403 (2025).
